# DRAC 2022: A public benchmark for diabetic retinopathy analysis on ultra-wide optical coherence tomography angiography images

**DOI:** 10.1016/j.patter.2024.100929

**Published:** 2024-02-08

**Authors:** Bo Qian, Hao Chen, Xiangning Wang, Zhouyu Guan, Tingyao Li, Yixiao Jin, Yilan Wu, Yang Wen, Haoxuan Che, Gitaek Kwon, Jaeyoung Kim, Sungjin Choi, Seoyoung Shin, Felix Krause, Markus Unterdechler, Junlin Hou, Rui Feng, Yihao Li, Mostafa El Habib Daho, Dawei Yang, Qiang Wu, Ping Zhang, Xiaokang Yang, Yiyu Cai, Gavin Siew Wei Tan, Carol Y. Cheung, Weiping Jia, Huating Li, Yih Chung Tham, Tien Yin Wong, Bin Sheng

**Affiliations:** 1 Shanghai Belt and Road International Joint Laboratory for Intelligent Prevention and Treatment of Metabolic Disorders, Department of Computer Science and Engineering, School of Electronic, Information, and Electrical Engineering, Shanghai Jiao Tong University, Department of Endocrinology and Metabolism, Shanghai Sixth People's Hospital Affiliated to Shanghai Jiao Tong University School of Medicine, Shanghai Diabetes Institute, Shanghai Clinical Center for Diabetes, Shanghai 200240, China; 2 MOE Key Laboratory of AI, School of Electronic, Information, and Electrical Engineering, Shanghai Jiao Tong University, Shanghai 200240, China; 3 Department of Computer Science and Engineering, The Hong Kong University of Science and Technology, Hong Kong 999077, China; 4 Department of Chemical and Biological Engineering, The Hong Kong University of Science and Technology, Hong Kong 999077, China; 5 Shanghai Sixth People's Hospital Affiliated to Shanghai Jiao Tong University School of Medicine, Shanghai 200233, China; 6 Tsinghua Medicine, Tsinghua University, Beijing 100084, China; 7 School of Electronic and Information Engineering, Shenzhen University, Shenzhen 518060, China; 8 VUNO, Inc., Seoul 06536, Korea; 9 AI/DX Convergence Business Group, KT, Seongnam 13606, Korea; 10 Johannes Kepler University Linz, Linz 4040, Austria; 11 School of Computer Science, Shanghai Key Laboratory of Intelligent Information Processing, Fudan University, Shanghai 200433, China; 12 Academy for Engineering and Technology, Fudan University, Shanghai 200433, China; 13 LaTIM UMR 1101, INSERM, 29609 Brest, France; 14 University of Western Brittany, 29238 Brest, France; 15 Department of Ophthalmology and Visual Sciences, The Chinese University of Hong Kong, Hong Kong 999077, China; 16 Department of Computer Science and Engineering, The Ohio State University, Columbus, OH 43210, USA; 17 Department of Biomedical Informatics, The Ohio State University, Columbus, OH 43210, USA; 18 Translational Data Analytics Institute, The Ohio State University, Columbus, OH 43210, USA; 19 School of Mechanical and Aerospace Engineering, Nanyang Technological University, Singapore 639798, Singapore; 20 Singapore Eye Research Institute, Singapore National Eye Centre, Singapore 168751, Singapore; 21 Centre for Innovation and Precision Eye Health; and Department of Ophthalmology, Yong Loo Lin School of Medicine, National University of Singapore, Singapore 119228, Singapore; 22 Ophthalmology and Visual Sciences Academic Clinical Program, Duke-NUS Medical School, Singapore 169857, Singapore; 23 School of Clinical Medicine, Beijing Tsinghua Changgung Hospital, Beijing 102218, China

## Abstract

We described a challenge named “DRAC - Diabetic Retinopathy Analysis Challenge” in conjunction with the 25th International Conference on Medical Image Computing and Computer Assisted Intervention (MICCAI 2022). Within this challenge, we provided the DRAC datset, an ultra-wide optical coherence tomography angiography (UW-OCTA) dataset (1,103 images), addressing three primary clinical tasks: diabetic retinopathy (DR) lesion segmentation, image quality assessment, and DR grading. The scientific community responded positively to the challenge, with 11, 12, and 13 teams submitting different solutions for these three tasks, respectively. This paper presents a concise summary and analysis of the top-performing solutions and results across all challenge tasks. These solutions could provide practical guidance for developing accurate classification and segmentation models for image quality assessment and DR diagnosis using UW-OCTA images, potentially improving the diagnostic capabilities of healthcare professionals. The dataset has been released to support the development of computer-aided diagnostic systems for DR evaluation.

## Highlights

• Provides the DRAC dataset, top-performing methods, and results
• Presents the deep learning methods in DR grading and lesion segmentation
• Summarizes the strategies for improving the model performance

## The bigger picture

Diabetic retinopathy (DR) is a common eye disease that can lead to visual impairment and even blindness. The study of DR is an important area that significantly affects the lives of millions of people worldwide. Understanding and managing DR is not only a medical challenge but also a societal one, emphasizing the need for early detection and intervention. A key to such understanding is ultra-wide optical coherence tomography angiography (UW-OCTA), a non-invasive imaging modality that could enable precise assessment of microvascular changes in retinal layers. To this end, we organized a medical image challenge and provided a UW-OCTA dataset for developing the computer-aided diagnostic system for DR diagnosis. The dataset can potentially accelerate the development of advanced artificial intelligence technologies and ultimately improve patient care.

## Graphical abstract

**Figure:**
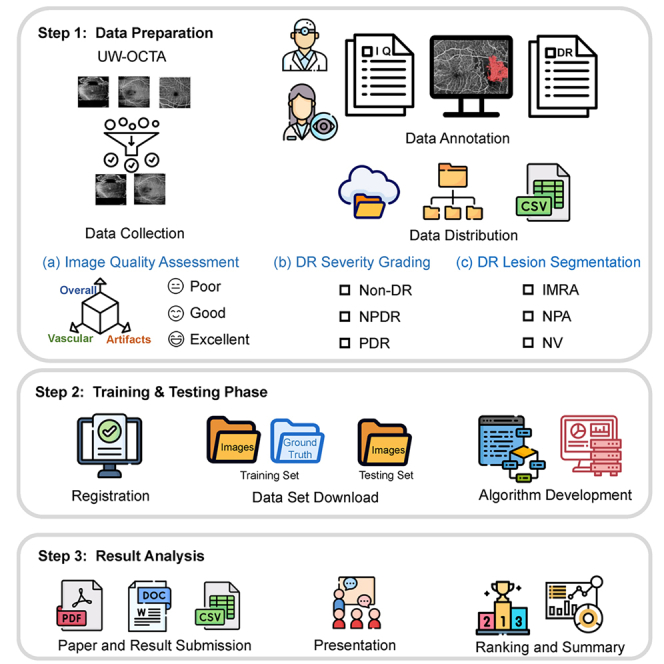

## Teaser

The DRAC challenge explored the use of artificial intelligence to tackle clinical tasks related to diabetic retinopathy (DR) using ultra-wide OCTA imaging. Here, the organizers present a comprehensive summary of the top three algorithms and the results for each task, including image quality assessment, lesion segmentation, and DR grading. These methods provide new insights into the diagnosis of DR and could potentially enhance the diagnostic capabilities of healthcare professionals in DR evaluation.

## Introduction

Diabetic retinopathy (DR) is one of the most common complications caused by diabetes.^1^ Patients with DR are more likely to get vision impairment and even blindness than healthy individuals. DR affects a large amount of the working-age population worldwide. According to the International Diabetes Federation,^2^ it is estimated that about 700 million people in the world are expected to have diabetes by 2045, and one-third of them will have DR. DR is diagnosed by visually inspecting retinal fundus images for the presence of retinal lesions, such as exudates, microaneurysm (MA), intraretinal microvascular abnormality (IRMA), and neovascularization (NV).^3^ Hence, the detection of these lesions is significant for DR diagnosis.

Regular DR screening and timely treatment can be implemented to reduce the risks of vision loss and blindness.^4^^,^^5^ However, there are many challenges to population screening. First, comprehensive DR screening puts a heavy burden on ophthalmologists. Especially in developing countries and rural parts, there may not be enough medical resources and ophthalmologists to perform the DR screening.^6^^,^^7^^,^^8^^,^^9^ Second, DR screening relies heavily on the experience of ophthalmologists. Differences in the experience of professional ophthalmologists may lead to different diagnoses, and the inadequate training of ophthalmologists can also result in misdiagnosis and low accuracy in DR screening.^8^^,^^10^ Third, systematic DR screening is associated with complicated social management and economic burden. Hence, the implementation of an efficient computer-aided system becomes indispensable in supporting manual DR screening. Such a system can assist in achieving precise diagnoses, thereby significantly alleviating the workload of ophthalmologists.^11^^,^^12^^,^^13^

The most commonly used imaging modalities for the clinical diagnosis of DR include fundus photography, fluorescein angiography (FA), and optical coherence tomography angiography (OCTA). Fundus photography is a common modality for rapid screening of DR. It effectively captures the distribution of hard exudates and retinal changes in severe non-proliferative DR (NPDR). However, it is difficult to detect early or small neovascular lesions. FA primarily detects the presence of NV but involves invasive fundus imaging and is unsuitable for patients with allergies, pregnancy, or poor kidney function. OCTA provides a non-invasive means of identifying changes in NV and assists ophthalmologists in diagnosing proliferative DR (PDR). Further, ultra-wide OCTA (UW-OCTA) reveals a broader peripheral retinal area beyond the scope of typical OCTA. For example, UW-OCTA imaging allows the assessment of peripheral retinal vascular networks, the detection of early stages of NPDR through capillary flow analysis, and the localization of NV lesions in patients with PDR.^14^^,^^15^ Several studies have used UW-OCTA images for DR diagnosis, screening, and follow-up purposes.^16^^,^^17^^,^^18^

In particular, Pichi et al.^18^ conducted a comparative analysis of UW-OCTA against UW-field FA (UWF-FA) and UWF color fundus photography (UWF-CP) for detecting NV in eyes with PDR. Their findings indicated that WF-OCTA can identify NV that is not evident in UWF-CP and serves as a swifter and safer alternative to UWF-FA for PDR monitoring, delivering comparable diagnostic accuracy. Khalid et al.^16^ conducted a retrospective observational case series comprising patients clinically diagnosed with PDR or severe NPDR. They reported that 12 × 12 mm UW-OCTA imaging exhibits superior PDR detection rates compared to clinical examination. This implies the non-invasive potential of this modality for early NV detection and characterization. Moreover, Kim et al.^19^ quantified the foveal avascular zone (FAZ), vessel density (VD), and NPA across three distinct OCTA field sizes: 3 × 3, 6 × 6, and 10 × 10 mm. Their experiment revealed that NPA measurements from the larger 10 × 10 mm scan were the sole discriminating parameter for the three NPDR stages, with the 10 × 10 mm scan demonstrating the highest sensitivity in determining five-grade DR severity. Similarly, Zhu et al.^20^ performed a prospective study comparing NV detection among four different OCTA field sizes: 3 × 3 mm angiography, 6 × 6 mm angiography, 15 × 9 mm montage, and 12 × 12 mm angiography. Both the 12 × 12 mm angiography scan and the 15 × 9 mm montage scanning exhibited high detection rates, but the former offered the advantage of taking less time to perform. All of these studies collectively underscore the potential of UW-OCTA imaging in DR detection.

Artificial intelligence challenges play a crucial role in advancing the application of deep learning techniques in medical image analysis. These challenges define one or more clinically significant tasks and provide the corresponding datasets, encouraging participants to develop algorithms for these tasks and enabling a fair comparison. Many challenges have been organized for DR analysis, such as ROC,^21^ IDRiD,^22^ and DeepDRiD,^23^ all employing fundus photography as the imaging modality. These initiatives have led to the creation of a multitude of state-of-the-art (SOTA) algorithms, significantly contributing to the research community. However, to our knowledge, there is a dearth of publicly available UW-OCTA datasets for evaluating DR. Against this background, we organized the Diabetic Retinopathy Analysis Challenge (DRAC) at the 25th International Conference on Medical Image Computing and Computer Assisted Intervention (MICCAI 2022), with the aim of establishing a benchmark and evaluation framework for the automated analysis of DR using UW-OCTA images.

In this paper, we describe in detail the DRAC dataset, challenge setup, and the top-performing solutions. We also report and analyze the challenge results, including ranking stability, model ensemble, and statistical significance. Finally, we discuss the clinical value of the dataset for future users, strategies to improve the model performance, limitations of the study, and the future work.

### Methods

#### Data

All the images in the DRAC dataset were acquired with the VG200D UW swept-source OCTA device. The scan captures a 12 × 12 mm area of the inner retinal layer, centered on the fovea. A total of 1,103 UW-OCTA images were collected with a resolution of 1,024 × 1,024 pixels. There are three types of annotations for corresponding clinically relevant tasks. First, image quality has a profound impact on disease diagnosis, and high-quality images are essential for accurate DR diagnosis. Therefore, the first task is image quality assessment, including poor, good, and excellent quality levels. The images of good and excellent quality can then be used for two other DR-related tasks: DR grading and DR lesion segmentation. One of the advantages of UW-OCTA imaging is its ability to detect NV, which is a critical indicator of PDR. Therefore, the second task is to identify PDR images from non-DR and NPDR images. In the lesion segmentation task, there are three different lesions to be segmented: IRMA, non-perfusion area (NPA), and NV. These three lesions are important morphological features of DR severity and can help to visualize pathological features of DR.^24^^,^^25^

In the process of image annotating, many factors affect the image quality,^26^^,^^27^ such as artifacts, vascular quality, etc. The specific annotation standard of image quality assessment is shown in Table 1. For DR grading, the fundus photograph corresponding to the UW-OCTA image was used to grade the DR, with the specific grading standard for non-DR, NPDR, and PDR referring to the international clinical DR severity scale.^28^ For each task, two ophthalmologists participated in the annotation process. First, the two ophthalmologists independently annotated the labels for each image according to the annotation standards. In the event of any disagreement, two additional, more experienced ophthalmologists were involved to help reach a consensus on the annotation.

**Table 1:** Detailed descriptions for each image quality level

Image quality level	Overall quality	Artifacts	Vascular quality
Poor	insufficient	severe	blurring
Good	moderate blurring or stripe noise	moderate	moderate blurring
Excellent	slight blurring or with slight stripe noise	slight	clear or slight blurring

The data division method for each task in this challenge is as follows. For image quality assessment task, the images were split into 60% for training (665 images) and 40% for testing (438 images). For the DR-grading task and the DR lesion segmentation task, the data division method was the same as the task of image quality assessment, but in the DR-grading task, we removed the images with the poor quality levels and images that caused considerable controversies about the DR grade by ophthalmologists, and then the rest of the images were retained as the training set (611 images) and the test set (386 images), respectively. In the DR lesion segmentation task, we only retained images that show representative lesions to form the training set (109 images) and the test set (65 images). The images were stored in gray png format. The ground truth of training set for the classification task was stored and provided in a CSV file. For the segmentation task, the ground truth was provided in the form of binary masks stored in png format.

#### Challenge setup

**Figure 1:**
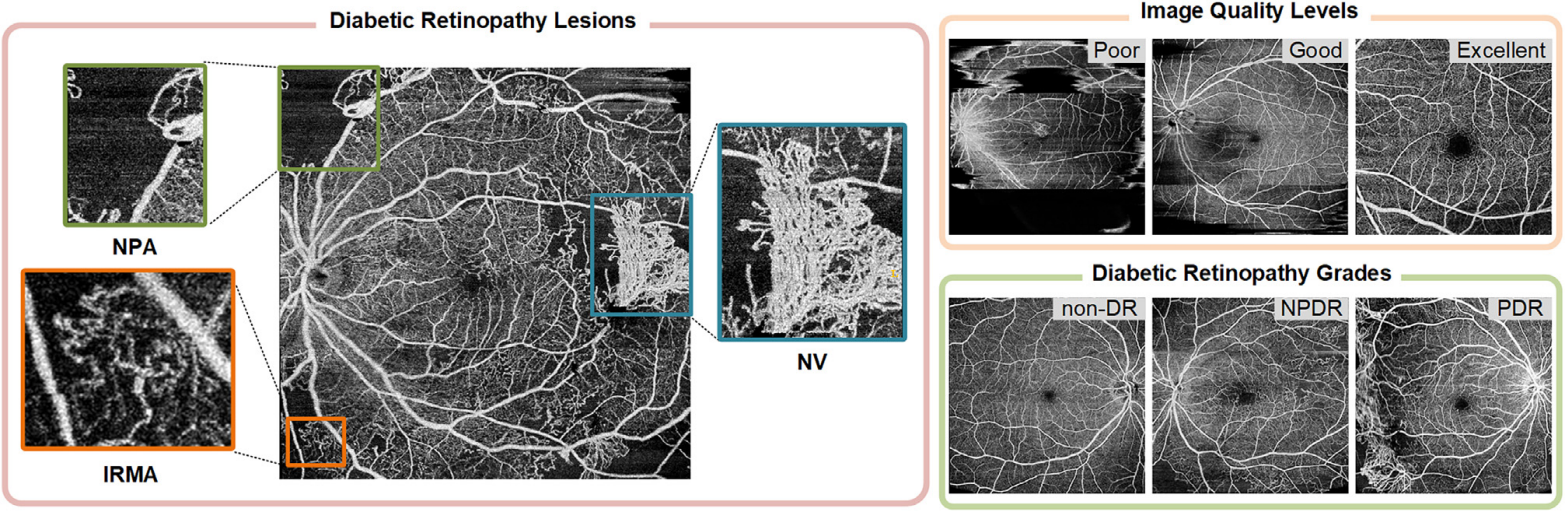
Examples of UW-OCTA images in three tasks of the challenge IRMA, intraretinal microvascular abnormality; NPA, nonperfusion area; NV, neovascularization; non-DR, non-diabetic retinopathy; NPDR, non-proliferative diabetic retinopathy; PDR, proliferative diabetic retinopathy.

The DRAC challenge aimed at providing a benchmark for evaluating the algorithms that are used for the automatic DR analysis using UW-OCTA images. It addressed the current lack of publicly available UW-OCTA datasets for fair performance evaluation of DR diseases. The challenge was subdivided into three tasks as follows. Image quality assessment and DR grading are both three-class classification tasks, so we have grouped them together as task 2 and task 3 in this challenge. Figure 1 shows the example images in each task.

(1) Task 1: segmentation of DR lesions. There are three types of lesions to be segmented, including IRMA, NPA, and NV.
(2) Task 2: image quality assessment. Classification of the image quality levels, including poor quality level, good quality level, and excellent quality level.
(3) Task 3: DR grading. Classification of DR grades according to the severity level of DR, including non-DR, NPDR, and PDR.

The organization of the challenge referred to the Biomedical Image Analysis Challenges guideline.^29^ The challenge was officially announced at MICCAI 2022 and was hosted on the Grand Challenge platform. The challenge website is available at https://drac22.grand-challenge.org. On the challenge website, the participants could have access to the dataset after they registered on the website and signed the challenge rule agreement. In addition, participants could browse the challenge rules and news, submit results, and find their rankings on the challenge website. In the case of multiple submissions, only the most recent run was counted for the final challenge result. We also provided submission guidelines for the participants. The challenge was launched in July 2022 by releasing the training dataset. The test set was released on August 8th, 2022, and the challenge submission was opened between August 8th, 2022, and September 12th, 2022. Each participating team was required to submit a method description paper with a minimum of 4 pages before October 8th, 2022. When participating in multiple tasks, each team could either submit several papers or a single paper reporting all methods. The details of the evaluation method can be seen in the supplemental information: evaluation metrics.

Finally, a total of 91 teams and individuals from more than 25 different countries or regions signed the challenge rule agreement consent form and downloaded the dataset throughout the challenge. Out of them, 17 teams submitted a total of 18 method description papers before the deadline, where 11, 12, and 13 different methods were reported for the three tasks, respectively. Some teams participated in two or more tasks and chose to report their methods in one single paper. During the satellite event at MICCAI 2022 on September 18th, 2022, we summarized the challenge results and invited the top-ranked teams to present their algorithms. The summary of the top three algorithms for each task is shown in Note S1. For a more in-depth description of a particular approach, please refer to MICCAI Challenge Proceedings.^30^

## Results

**Figure 2:**
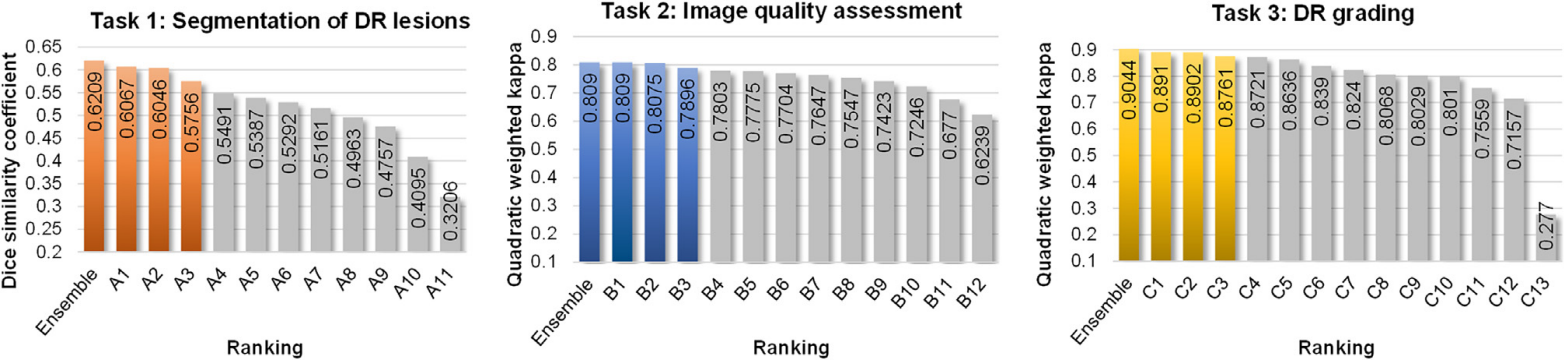
Bar charts of the final rankings for three tasks The colored bars show the ensemble and the top three scores in each task. Ensemble represents the ensemble results of the top three algorithms.

The final rankings of the three tasks are shown in Figure 2. To facilitate clarity, we adopt the labels A, B, and C to denote the algorithms corresponding to task 1 (DR lesion segmentation), task 2 (image quality assessment), and task 3 (DR grading), respectively, followed by a number indicating the ranking of the algorithm in this task. For example, A1, B1, and C1 represent the first-ranked algorithms in task 1, task 2, and task 3, respectively. We first present the results obtained by the participating teams and then analyze the ranking stability of these algorithms. We also present the ensemble of the results of the top three algorithms in each task and report the statistical significance analysis of the algorithms.

### Task 1: Segmentation of DR lesions

In the task of DR lesion segmentation, out of the total 24 teams that submitted the results on the test set, 11 teams submitted method description papers. The average dice similarity coefficient (DSC) of the top ten teams ranged from 40.95% to 60.67%. The DSC distribution of each class of lesion is shown in Figure S1. Among these three lesions, IRMA has the lowest segmentation performance, with DSCs ranging from 29.53% to 47.04% for the top ten algorithms. NPA has the highest segmentation performance, with DSCs ranging from 46.59% to 69.26% for the top ten algorithms, and NV ranks second, with DSCs ranging from 46.73% to 65.71% for the top ten algorithms. The complex lesion features could explain the low segmentation performance of IRMA because IRMAs are usually thin vessels that spread throughout the image. In addition to the DSC used in the challenge, we also use sensitivity (SEN), precision (PRE) and specificity (SPE) to evaluate the segmentation performance of each method. The quantitative results of the top three methods are shown in Table S1.

### Task 2: Image quality assessment

**Figure 3:**
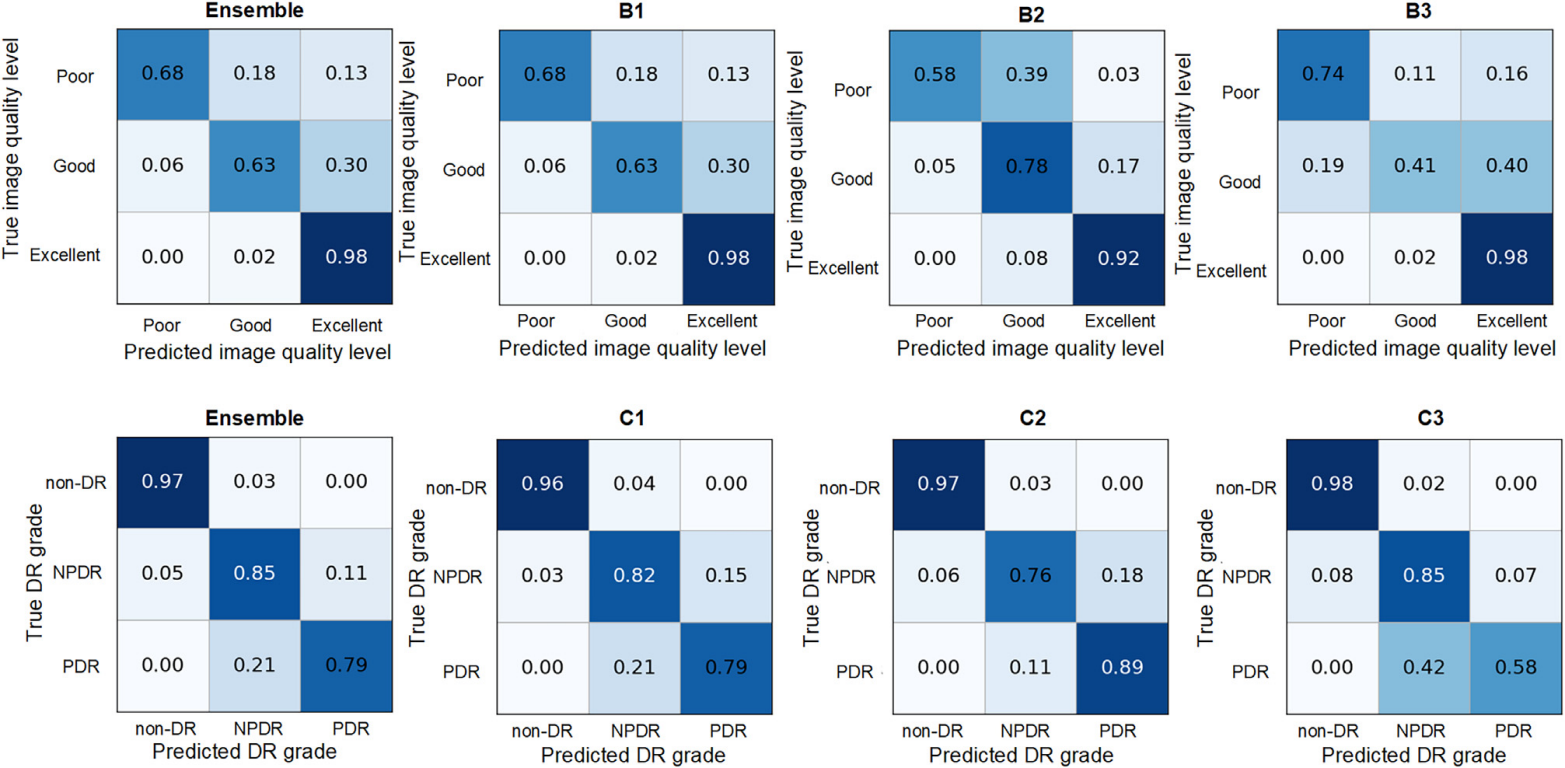
Confusion matrix for image quality assessment task and DR-grading task The top row is the image quality assessment task and the bottom row is the DR-grading task. From left to right are the ensemble and first-, second-, and third-ranked results, respectively. Ensemble represents the ensemble results of the top three algorithms.

In the task of image quality assessment, a total of 45 teams submitted results, and 12 teams submitted method description papers, where the quadratic weighted kappa (QWK) of the top ten teams ranged from 0.7246 to 0.8090. Table S2 shows the quantitative results of the top three algorithms for the metrics of sensitivity, specificity, and F1 score. Combined with the confusion matrices of the top three methods in Figure 3, there are two notable observations. Firstly, there is a tendency to misclassify some images as having excellent quality regardless of whether their actual quality is good or poor. This phenomenon can be attributed to the data imbalance of the dataset, where 80% of the images have an excellent quality level, while the remaining 20% have poor and good quality levels. This imbalance tends to bias the training toward the majority class, as the models strive to minimize the loss function. Although techniques like resampling can improve the classification performance of the minority class, they cannot fully compensate for the performance gap between the minority and majority classes caused by the data imbalance. Secondly, another noticeable trend is the misclassification of images between the adjacent classes, where some images with a poor quality level are misclassified as good quality level while some images with a good quality level are misclassified as excellent quality level. This can be explained by subtle feature distinctions between adjacent image quality levels, making it difficult for the network to classify correctly. Combined with the data imbalance issue, we observe that, for example, in ensemble results, 30% of the images with good image quality are misclassified as excellent image quality.

### Task 3: DR grading

In the task of DR grading, a total of 45 teams submitted results, and 13 teams submitted method description papers, where the quadratic weighted kappa of the top ten teams ranged from 0.7157 to 0.8910. Table S3 shows the quantitative results of the top three algorithms for the metrics of sensitivity, specificity, and F1 score. Combined with the confusion matrices of the top three methods in Figure 3, we can see that the non-DR class achieves the highest accuracy, while the misclassifications mainly occur between the NPDR and PDR classes. Apart from the influence of the data imbalance, where the non-DR, NPDR, and PDR account for approximately 55%, 35%, and 10% of the dataset images, respectively, another possible factor is that the NV regions, which are a representative indicator of PDR, are usually small in size, posing a challenge for the classification network to accurately detect these areas, thereby leading to misclassifications between NPDR and PDR.

### Ranking stability

Inspired by the challengeR toolkit,^31^ we performed bootstrapping (1,000 bootstrap samples) to assess the stability of rankings with respect to sampling variability. To quantitatively assess the ranking stability, the agreement of the challenge ranking and the ranking of each bootstrap on the test set was determined via Kendall’s τ, which is a rank correlation coefficient with a value between −1 (reverse ranking order) and 1 (identical ranking order). The violin plots shown in Figure S3 illustrate the bootstrap results for each task. We obtained Kendall’s τ of 0.9313, 0.7697, and 0.8802 for the tasks of DR lesion segmentation, image quality assessment, and DR grading, respectively. Figure S4 shows a blob plot of the bootstrap rankings for each task.

### Ensemble of top three algorithms

The ensemble of networks has shown great power in improving model performance.^32^^,^^33^^,^^34^ Thus, it is interesting to explore the ensemble of the top three methods in each task. For the segmentation task, we use three forms of ensemble to generate the final segmentation output, including logical AND, logical OR, and majority voting. The DSCs of the three forms of ensemble results are 47.10%, 45.36%, and 49.95% for IRMA, 67.91%, 66.83%, and 68.78% for NPA, and 63.33%, 60.23%, and 67.55% for NV. For each of the three classes, the highest DSC is achieved by the majority voting strategy, of which the averaged DSC is 62.09%—which is 1.42% higher than the best result of the participating algorithms. The detailed performance of the ensemble result with a majority voting is shown in Table S1. For the two classification tasks, we use a majority voting strategy from the top three results to generate the ensemble result. For image quality assessment, the quadratic weighted kappa of the ensemble result is 0.8090, which is equal to the best score of participating algorithms. The detailed performance of the ensemble result is shown in Table S2. For DR grading, the quadratic weighted kappa of the ensemble result is 0.9044, which is 1.4% higher than the best score of participating algorithms. The detailed performance of the ensemble result is shown in Table S3. From the ensemble results in both the segmentation task and the classification task, we can see that the ensemble of the model has great power in improving the performance of deep learning methods.

### Statistical significance analysis of the algorithms

For each task of the challenge, the statistical comparison of the score of each team is done with the one-tailed Wilcoxon signed rank test at a 5% significance level. The challengeR toolkit^31^ is used to perform the significance analysis and generate the significance map. For the task of DR lesion segmentation, the significance maps in Figure S2 show the results of the statistical significance analysis for the three lesions. In conclusion, there is a significant difference between multiple algorithm pairs for IRMA segmentation, but for the segmentation of NPA and NV, only a few pairs of algorithms show significant differences. For the two classification tasks, the statistical comparison of the score of each team is shown in Figure S5. In the task of image quality assessment, there are no significant differences among B1, the ensemble model, and B2. In the task of DR grading, the ensemble model is significantly superior to the algorithms C1, C2, and C3, but there is no significant difference between C1 and C2.

## Discussion

### Clinical value of the dataset for future users

UW-OCTA imaging offers significant advantages in the monitoring and management of DR. First, compared to OCTA images, UW-OCTA can provide a wider field of view, allowing ophthalmologists to detect more abnormal lesions and microvascular changes. Second, compared to fundus photography and FA, OCTA can provide more comprehensive and detailed information about the retinal vascular structure in a non-invasive manner. OCTA does not require the use of contrast agents, which is an important advantage for patients who are allergic or intolerant to contrast agents. In contrast, FA involves injecting contrast agents into the patient’s body, which can lead to allergic reactions or other discomfort. Moreover, OCTA can capture tiny blood vessels, allowing ophthalmologists to detect microvascular lesions and microvascular occlusions earlier, facilitating timely intervention.

We have released the largest UW-OCTA dataset (DRAC dataset) with corresponding annotations for three DR-related tasks: image quality assessment, DR lesion segmentation, and DR grading. Firstly, the image quality assessment task helps ensure the reliability of retinal images obtained from OCTA devices. This is crucial for accurate disease diagnosis, as diagnoses based on poor-quality images can lead to erroneous judgments. Through this task, we can raise the standards of medical image acquisition and ensure that healthcare professionals and researchers have high-quality data for further analysis and diagnosis. Secondly, the DR lesion segmentation task provides automated segmentation tools for different types of DR lesions, including IRMA, NPA, and NV. This means that ophthalmologists and researchers can identify lesion areas more quickly and accurately, enabling earlier intervention and treatment. This is vital for reducing the risk of blindness and improving the quality of life for patients. Finally, the DR-grading task helps to classify the severity of DR in patients, with particular emphasis on the detection of PDR, given the high detection rate achieved using OCTA images. This will help ophthalmologists to develop more accurate treatment plans and monitor the progression. We believe that this dataset can provide valuable resources for the research community and pave the way for the development of artificial intelligence algorithms.

### Strategies to improve the model performance

We summarize the characteristics of the top three competing solutions, as shown in Tables 2 and S4, and then recognize a selection of frequently employed strategies with the potential to improve algorithmic performance.

**Table 2:** Summary of model architecture, loss function, and optimizer from top three algorithms in each task of the challenge

	Algorithm	Model architecture	Loss function	Optimizer	Score
Task 1	A1	U2Net	WDL + FL/CE	AdamW	0.6067
	A2	ConvNext + SegFormer + Swin Transformer	DL + FL/CE	AdamW	0.6046
	A3	nnUNet	DL + CE	SGD	0.5756
Task 2	B1	EfficientNet	Smooth L1	AdamW	0.8090
	B2	BEIT + NFNet	CE	AdamW	0.8075
	B3	Inception-V3 + SE-ResNeXt + ViT	CE	SGD	0.7896
Task 3	C1	EfficientNet	Smooth L1	AdamW	0.8910
	C2	BEIT	CE	AdamW	0.8902
	C3	DenseNet121 + Efficientnet	CE	Adam	0.8761

In the score column, task 1 utilizes the DSC score, while tasks 2 and 3 employ QWK scores. DL, dice loss; WDL, weighted dice loss; FL, focal loss; CE: cross-entropy; SGD, stochastic gradient descent.

For the data preprocessing and data augmentation, when considering input image resolution, most teams choose to maintain the original image size to prevent any loss of features. Regarding image normalization, the leading teams often opt for scaling pixel values within the 0–1 range or implementing zero-mean unit variance normalization. Such normalization of input image pixel values can help accelerate the convergence speed of the model, mitigating issues like vanishing and exploding gradients to enhance training efficiency. Additionally, it can maintain stable numerical computations and enhance overall model performance. All teams used some form of data augmentation to enhance their models. The most popular image transformation is flipping, which was adopted by all top three teams. Other commonly used image transformations include rotation, scaling, brightness modification, contrast modification, etc. In addition, more complex multi-image data augmentation techniques are also effective for improving model performance, including MixUp and CutMix.

For the network architecture, despite numerous segmentation networks having been proposed in recent years, the classical U-Net-like architectures, such as U2Net and nnUNet, remain highly competitive in segmentation tasks. This finding is in accordance with the winning methods in other recent segmentation challenges.^35^^,^^36^^,^^37^ In the classification task, EfficientNet has been used by many teams and exhibited remarkable performance. Additionally, ensemble methods were widely adopted by the leading teams, although with varying ensembling strategies. One method involves integrating predictions from distinct network architectures, while another incorporates predictions generated by the identical network architecture but fed with multiple transformed versions of the same inference image as input. In terms of accuracy, both ensemble strategies have the potential to achieve SOTA performance.

For the loss function, the dice coefficient is used as the evaluation metric in the segmentation task, with all three leading teams using the dice loss to supervise learning. This is consistent with the previous research, which demonstrated the effectiveness of the metric-sensitive loss functions in improving the corresponding metric scores.^38^ In addition, a combination of different loss functions is effective in improving model performance, with the dominant combinations including dice loss paired with either cross-entropy loss or focal loss. In terms of the optimizer, AdamW, which performs L2 regularization for larger weights to further improve the training of the model, was the most commonly used by these teams in both segmentation and classification tasks.

For the post-processing, in disease-grading tasks, segmentation of the lesion area can help improve the performance of disease grading. For instance, in DR grading, the presence of NV corresponds to the PDR grade. However, in certain images, NV regions are often small and are difficult to detect, potentially leading the model to misclassify the image as NPDR. In such cases, if a lesion segmentation model has detected the NV region, the image can be corrected to PDR grade. The top two methods in the DR-grading task both leveraged the lesion segmentation results to correct the predicted DR grade made by the classification model, resulting in an improvement in classification performance.

### Limitations of the study

In the domain of DR diagnosis, PDR stands as the most severe grade, carrying a high risk of causing severe vision impairment and even blindness. The employment of UW-OCTA imaging has demonstrated a superior PDR detection rate in comparison to clinical examination.^16^ Consequently, a substantial pool of PDR images for training becomes essential for deep learning methods to extract generalized features of PDR lesions. However, within this challenge dataset, only about 11% (70 images) fall into the PDR category in the training set, hindering the ability of the methods to extract effective PDR features and achieve generalization in clinical practice. In addition, the performance of the NPA segmentation still lags behind that reported in some existing studies. This performance gap needs to be further investigated from three primary angles. First, larger datasets, as used in some studies,^39^^,^^40^ may improve model performance. Second, including FAZ in NPA segmentation, as seen in these works,^39^^,^^40^^,^^41^ is crucial. The fixed position and distinct patterns of FAZ in the image simplify its segmentation, thereby improving the overall segmentation performance. Finally, incorporating a variety of retinal layers into the network,^39^^,^^40^^,^^41^ particularly by integrating data from multiple retinal layers such as the superficial vascular complex, intermediate capillary plexus, and deep capillary plexus, allows the network to extract richer features, further improving the accuracy of NPA segmentation.

Moreover, our dataset does not contain meta-information about the images, such as age, gender, height, and medical history. This lack prevents us from providing statistics on the number of eyes and patients within the dataset. In addition, the Grand Challenge platform deployed in this challenge offers two submission options: one is algorithm submission and another is result submission. In comparison, result submission boasts the advantage of being free for organizers, consuming fewer computational resources of the platform, and presenting a simple online evaluation process for participants. Thus, we have opted for result submission as the avenue for our challenge submission. Nonetheless, a notable drawback lies in the fact that participants could have access to the test images without annotations. This opens the door for participants to optimize network parameters using the test images, potentially introducing biases into the output of the algorithm.

### Future work

In our future efforts for the DRAC challenge, we intend to expand the dataset, providing more images for model training and validation. Additionally, we expect to include meta-information that can facilitate subgroup analysis, enabling us to assess model biases across different populations, such as gender and age. Moreover, we will make the DRAC dataset accessible to the community through the DRAC website, with the hope that it will prove highly valuable for researchers addressing various topics in this field. Beyond the end of this challenge, we will also maintain an open post-challenge submission system, see in https://drac22.grand-challenge.org/post-challenge-submission, encouraging the evaluation of innovative solutions and driving progress in the domain of automated DR analysis.

### Conclusion

The DRAC challenge held at MICCAI 2022 provides a benchmark DRAC dataset and evaluation framework for automatic DR analysis from UW-OCTA images, including the tasks of DR lesion segmentation, image quality assessment, and DR grading. With numerous participants from academia and industry worldwide, the challenge offers diverse solutions and comparable diagnostic results to benefit ophthalmologists engaged in DR analysis. These solutions are described in detail in the MICCAI Challenge Proceedings, and many teams, including the top three teams in each task, have open-sourced their code, which can significantly accelerate methodological developments in the research community. We thoroughly summarized and discussed the algorithms and results from participating teams in this paper. These algorithms hold the potential to be integrated into future computer-assisted automatic diagnostic systems for DR, which can help reduce the burden on healthcare workers and improve the accuracy of DR diagnosis. Nevertheless, ongoing research efforts are still needed to improve the model and realize a clinically applicable diagnostic system for DR. To date, the challenge website remains open for post-challenge submissions, with the aim of providing a sustainable benchmarking and evaluation platform for the research community.

## Experimental procedures

### Resource availability

#### Lead contact

Requests for further information and resources should be directed to the lead contact, Bin Sheng (shengbin@sjtu.edu.cn).

#### Materials availability

This study did not generate any new materials.

#### Data and code availability

The DRAC dataset has been deposited to the Zenodo data repository under https://doi.org/10.5281/zenodo.10280358.^42^ The code for methods A1, B1, and C1 has been archived at Zenodo under https://doi.org/10.5281/zenodo.10254200.^43^ The code for methods A2, B2, and C2 has been archived at Zenodo under https://doi.org/10.5281/zenodo.10212156.^44^ The code for method A3 has been archived at Zenodo under https://doi.org/10.5281/zenodo.10254707.^45^ The code for method B3 has been archived at Zenodo under https://doi.org/10.5281/zenodo.10210181.^46^ The code for method C3 has been archived at Zenodo under https://doi.org/10.5281/zenodo.10209637.^47^

### Ethics statement

The study adhered to the guidelines of the Helsinki Declaration and had the approval of the Ethics Committee of Shanghai Sixth People’s Hospital(2019-KY-052(K)-(3)), Shanghai, China. All patients signed written informed consent for participation. According to the Common Rule by the Department of Health and Human Services, this informed consent includes that eight elements of information about the research study be provided to the patient or his or her legally authorized representative.^48^ These elements include a statement that the study is investigational along with a description of the research and its objectives, a description of foreseeable risks, a description of foreseeable benefits to participants as well as to others, information about reasonable alternatives, a statement clarifying the implications of research participation for the subject’s confidentiality, a statement about compensation if injury occurs (for investigations involving more than minimal risk), information about how the subject can obtain answers to pertinent research questions, and a statement about the voluntary nature of study participation and the subject’s right to withdraw.

### Evaluation metrics

There are three leaderboards in the challenge website, and each task corresponds to a leaderboard. In the task of DR lesion segmentation, DSC is used for the algorithm evaluation and ranking in segmentation task. In case of a tie, Intersection of Union (IoU) is used as an auxiliary ranking metric. The DSC and IoU are calculated as follows.

(Equation 1) $$\text{DSC}=\frac{2\left|G\cap P\right|}{\left|G\right|+\left|P\right|}$$

(Equation 2) $$\text{IoU}=\frac{\left|G\cap P\right|}{\left|G\cup P\right|}$$

where *G* and *P* are the ground-truth mask and predicted mask, respectively. The metric is calculated for each class independently, and the results are then averaged.

For the tasks of image quality assessment and DR grading, quadratic weighted kappa is used for the algorithm evaluation and ranking. In case of a tie, area under the receiver operating characteristic curve (AUC) is used as an auxiliary ranking metric. Considering that this is a multi-class task, the macro averaging^49^ and one-vs.-one (OVO) strategy^50^ are used to calculate the AUC value. Macro averaging calculates the AUC value for each label and finds their unweighted mean. The OVO strategy computes the average AUC of all possible pairwise combinations of classes. The quadratic weighted kappa $K_{w}$ is calculated as follows.

(Equation 3) $$K_{w}=1-\frac{\sum_{i,j}w_{i,j}O_{i,j}}{\sum_{i,j}w_{i,j}E_{i,j}}$$

where *O* and *E* are the histogram matrix and expected matrix, respectively, with the size of N×N. The weighted matrix w is defined by $w_{i,j}=\frac{{\left(i-j\right)}^{2}}{{\left(N-1\right)}^{2}}$, where *i* and *j* denote the actual value and the predicted value, respectively. N is the number of classes.

## Supplemental information

Document S1. Figures S1–S5, Tables S1–S5, and Notes S1 and S2

Document S2. Article plus supplemental information
