# Supplementary material for: DRAC 2022: A public benchmark for diabetic retinopathy analysis on ultra-wide optical coherence tomography angiography images
Source: Patterns (N Y). 2024 Feb 8;5(3):100929. doi: 10.1016/j.patter.2024.100929 (PMC10935505; doi:10.1016/j.patter.2024.100929)
Supplement: Document S1. Figures S1–S5, Tables S1–S5, and Notes S1 and S2 [file mmc1.pdf]

Patterns, Volume *5*

Supplemental information

DRAC 2022: A public benchmark for diabetic retinopathy analysis on ultra-wide optical coherence tomography angiography images

Bo Qian, Hao Chen, Xiangning Wang, Zhouyu Guan, Tingyao Li, Yixiao Jin, Yilan Wu, Yang Wen, Haoxuan Che, Gitaek Kwon, Jaeyoung Kim, Sungjin Choi, Seoyoung Shin, Felix Krause, Markus Unterdechler, Junlin Hou, Rui Feng, Yihao Li, Mostafa El Habib Daho, Dawei Yang, Qiang Wu, Ping Zhang, Xiaokang Yang, Yiyu Cai, Gavin Siew Wei Tan, Carol Y. Cheung, Weiping Jia, Huating Li, Yih Chung Tham, Tien Yin Wong, and Bin Sheng

Supplemental information

DRAC 2022: A Public Benchmark for Diabetic Retinopathy Analysis on Ultra-Wide Optical Coherence Tomography Angiography Images

Bo Qian, Hao Chen, Xiangning Wang, Zhouyu Guan, Tingyao Li, Yixiao Jin, Yilan Wu, Yang Wen, Haoxuan Che, Gitaek Kwon, Jaeyoung Kim, Sungjin Choi, Seoyoung Shin, Felix Krause, Markus Unterdechler, Junlin Hou, Rui Feng, Yihao Li, Mostafa El Habib Daho, Dawei Yang, Qiang Wu, Ping Zhang, Xiaokang Yang, Yiyu Cai, Gavin Siew Wei Tan, Carol Y. Cheung, Weiping Jia, Huating Li, Yih Chung Tham, Tien Yin Wong, Bin Sheng

Supplemental Note S1: Summary of top three solutions

Task 1: segmentation of DR lesions

A1 (Kwon et al.) A1[1] proposed a method for DR lesion segmentation based on the U2-Net network[2]. They developed two independent U2-Net models for different lesions. One model focused on imbalanced data settings for small lesions (IRMA and NV), while the other emphasized hard example mining for NP segmentation. To prepare the input image, they normalized the pixel values to the range [0,1] and used the original resolution of 1024 × 1024 pixels as the network input. For NP segmentation, they employed a combination of weighted dice loss and auxiliary focal loss[3] as the training loss function, which effectively penalized false positive pixels and hard-to-distinguish pixels. On the other hand, for small-lesion segmentation, they used weighted dice loss and auxiliary binary cross-entropy loss during training, with a milder penalty on false positive pixels compared to the focal loss. During the test phase, they used Test-Time Augmentation (TTA) with rotation transformations for both IRMA and NV. In the case of NPA, they averaged the predictions of five independent models, each trained with a different random number seed. They also applied TTA with rotation transformations to each of these models. Post-processing techniques were used to obtain the final lesion predictions. For NP, they applied a dilation operation with a kernel size of 5 to the predicted NPA masks. For IRMA and NV masks, if a pixel was predicted to belong to both IRMA and NV, they retained the class with higher confidence. Code is available at https://github.com/vuno/DRAC22_MICCAI_FAI and has been archived at Zenodo under https://doi.org/10.5281/zenodo.10254200.[4]

A2 (Choi et al.) A2[5] introduced an ensemble model by combining ConvNext[6], SegFormer[7], and Swin Transformer[8] to develop their segmentation model. For ConvNext, they employed a combination of dice loss and cross-entropy loss during training, while for SegFormer and Swin Transformer, the combination of dice loss and focal loss was used. To improve segmentation performance, colorization and contrast limited adaptive histogram equalization (CLAHE)[9] techniques were applied. The original image size of 1024 × 1024 was retained to prevent feature loss during training. The optimization process involved using AdamW[10] as the optimizer and polyLR for learning rate adjustment strategy. Additionally, the best-performing segmentation network was utilized to generate pseudo-labels for the images in Task 3 of DR grading. These images, along with their pseudo-labels, were used as input again for the network training. Finally, the segmentation results for IRMA and NV were obtained by applying an ensemble of the three networks using the morphological logical OR operation, while the segmentation results of NPA were obtained using the logical AND operation. Code is available at https://github.com/KT-biohealth/DRAC22 and has been archived at Zenodo under https://doi.org/10.5281/zenodo.10212156.[11]

A3 (Krause et al.) A3[12] employed nnU-Net framework[13] as their segmentation model, training individual models for each class, which allowed to fine-tune and experiment specifically for each class. Throughout the training process, they utilized a combination of dice loss and cross entropy loss, using the SGD optimizer with a momentum of 0.99 and a polynomial learning rate scheduler. To ensure generalization, they applied more than 10 data augmentation techniques for the IRMA and NV classes. However, for NPA, they discovered that fewer augmentation techniques, including flip, scale, and rotation, yielded superior results. The network was trained for 200 epochs, and a 5-fold cross-validation approach was adopted. Subsequently, only the best two or three folds were ensembled to avoid negatively impacting the overall Dice score. For IRMA, they achieved better predictions by ensembling the results using the union of all predictions. This approach proved advantageous in mitigating false negative pixels associated with IRMA. As for NPA and the NV classes, a majority vote ensembling strategy was employed to generate the final predictions. Code is available at https://github.com/flixmk/DRAC22-JKU and has been archived at Zenodo under https://doi.org/10.5281/zenodo.10254707.[14]

Task 2: image quality assessment

B1 (Kwon et al.) B1[1] employed EfficientNet-b2[15] as the network architecture, initializing it with pre-trained weights from ImageNet. To address class correlation, they treated the classification task as a regression problem in which the regression labels are 0, 1 and 2 for the three classes, respectively. Further, they used the class-specific operating thresholds to produce final predictions, as shown in Equation 4, where $\hat{y}$ represents the prediction given the input x, and f_{reg} represents the regression model. To improve generalization, they used various augmentation techniques, including flip, random gamma, and sharpen. During network training, they utilized the Smooth L1 loss function and the AdamW optimizer with a fixed learning rate of 0.0002, opting not to employ a scheduler. The model underwent 150 epochs of training with a batch size of 8. During the testing phase, a five-fold cross-validation approach was used, and ensemble methods were utilized by averaging the predictions generated by the models within each fold. Additionally, they applied TTA with flip operators for each model to further improve classification performance. Code is available at https://github.com/vuno/DRAC22_MICCAI_FAI and has been archived at Zenodo under https://doi.org/10.5281/zenodo.10254200.[4]

$$\hat{y} = \begin{cases} 0, & \text{if } f_{\text{reg}}(x) < 0.54 \\ 1, & \text{if } 0.54 \leq f_{\text{reg}}(x) < 1.5 \\ 2, & \text{otherwise} \end{cases} \tag{1}$$

B2 (Choi et al.) B2[5] employed BEIT[16] and NFNet[17] to develop the classification model. They preprocessed the input images by dividing each pixel value by 255, and used both vertical and horizontal flipping as data augmentation. They utilized the cross-entropy loss function, AdamW optimizer and the learning rate scheduler of StepLR[16] during the training process. The final result was achieved by an ensemble of BEIT and NFNet models using the following equation:

$$P = 0.55 \times S(f_{BEIT}) + 0.45 \times S(f_{NFNet}) \tag{2}$$

where P is the probabilities of the predicted classes, S represents the softmax function, and f_{BEIT} and f_{NFNet} denote the outputs from the BEIT and NFNet networks, respectively. Code is available at https://github.com/KT-biohealth/DRAC22 and has been archived at Zenodo under https://doi.org/10.5281/zenodo.10212156.[11]

B3 (Hou et al.) B3[18] designed an ensemble comprising three models: Inception-V3[19], SE-ResNeXt[20], and Vision Transformer (ViT)[21]. To address the challenge of overfitting due to limited samples, the models were pre-trained on the OCTA-25K-IQA-SEG dataset[22]. Then fine-tuning was carried out on the DRAC dataset, incorporating effective data augmentation techniques like MixUp[23] and CutMix[24], etc. All images were resized to specific dimensions: 224 × 224 for SE-ResNeXt, 384 × 384 for ViT, and 512 × 512 for Inception-V3. Additionally, they normalized all images by setting the mean to 0.5 and standard deviation to 0.5. For training the networks, they employed the SGD algorithm with an initial learning rate of 0.001 and the cosine annealing learning rate schedule. Each network was trained for 100 epochs. For the ensemble, the final prediction for each OCTA image was generated by averaging the outputs of the individual models. Code is available at https://github.com/FDU-VTS/DRAC and has been archived at Zenodo under https://doi.org/10.5281/zenodo.10210181.[25]

Task 3: DR grading

C1 (Kwon et al.) C1[1] utilized EfficientNet-b2[15] as their network architecture, initializing it with pre-trained weights from ImageNet. They approached the classification task as a regression problem, where the predicted class was obtained by rounding the predicted value to the nearest integer. During network training, they applied the Smooth L1 loss function and the AdamW optimizer with a fixed learning rate of 0.0002. The model was trained for 150 epochs with a batch size of 8. During the testing phase, a five-fold cross-validation approach was used, and ensemble methods were utilized by averaging the predictions generated by the models within each fold. Additionally, they applied TTA with flip operators for each model to further improve classification performance. In terms of post-processing, they found that the DR grading model tended to ignore the NV lesion, which is a clear indicator of PDR. As a result, the model sometimes misclassified PDR as NPDR. Therefore they corrected the misclassified NPDR predictions with PDR if the NV lesion was present in the segmentation model's prediction mask. Conversely, if the segmentation model did not predict a lesion, they corrected the DR grading model predictions to normal. This post-processing pipeline demonstrated the effectiveness of improving the classification performance of their deep learning system. Code is available at https://github.com/vuno/DRAC22_MICCAI_FAI and has been archived at Zenodo under https://doi.org/10.5281/zenodo.10254200.[4]

C2 (Choi et al.) C2[5] utilized the BEIT network to develop their classification model. Various image processing techniques such as image coloring and resampling were applied to the dataset prior to training. The resampling involved randomly processing images of the minority class, including flipping, rotating, zooming, sharpening, and then repeatedly copying them until this class had the same number of images as the majority class. During training, the image was normalized by dividing each pixel value by 255, and the data was augmented by the flip operation. Also, the cross-entropy loss function, the AdamW optimizer and the learning rate scheduler of StepLR[16] were used. For the post-processing, they designed a combination of classification and segmentation results to improve the classification performance of DR grading. In this approach, each image underwent segmentation for three lesions: IRMA, NPA, and NV, using the segmentation model. The probabilities for each class were then obtained through the DR grading classification model. A specific rule was used to refine the classification results based on the segmentation results. For example, if the NV area from the segmentation model was large and the probability of PDR from the classification model was high, the final classification result would be set as PDR. Additionally, if the initial classification result was normal but the area of IRMAs was large, the final result would be changed to NPDR. If no lesion masks were present in the segmentation result, the final classification result would be set as normal. Code is available at https://github.com/KT-biohealth/DRAC22 and has been archived at Zenodo under https://doi.org/10.5281/zenodo.10212156.[11]

C3 (Li et al.) C3[26] carried out a comprehensive evaluation of the classification performance for DR grading, using19 different backbones with the ImageNet pre-trained weights. During the training process, the original image size of 1024 ×

1024 was used as input to the network, and the cross entropy was chosen as the loss function. Various data augmentation techniques, including random crop, random flip, and random rotation, were applied, along with the Adam optimizer with an initial learning rate of 0.0001 and a weight decay of 0.0001. The ExponentialLR learning strategy schedule was used with a gamma value of 0.99. Training was performed for 1000 epochs with a batch size of 4. Five-fold cross-validation was performed on each of the 19 backbones to ensure robust evaluation. Based on kappa values from the test set, the two most best-performing folds from the DenseNet121[27] and Efficientnet-b3[15] backbones were selected and fine-tuned using the entire training set. Finally, the model ensemble for DenseNet121 and Efficientnet-b3 was implemented and achieved the best classification results. Code is available at https://github.com/Mostafa-EHD/Diabetic_Retinopathy_OCTA and has been archived at Zenodo under https://doi.org/10.5281/zenodo.10209637.[28]

Supplemental Note S2: Results of the participating teams

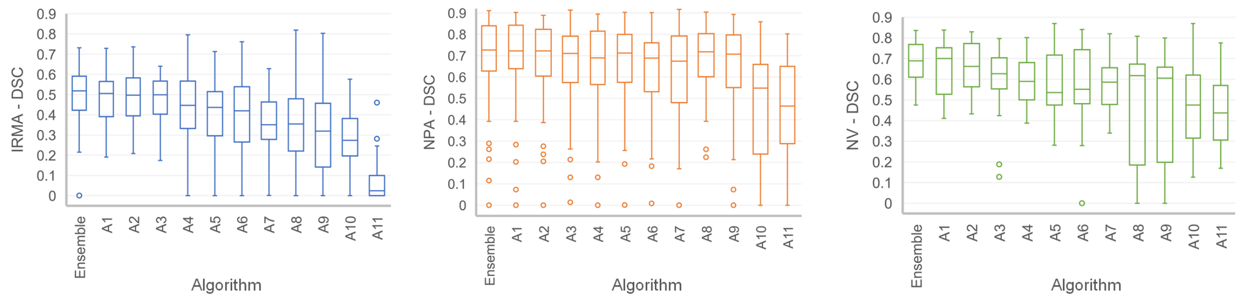

Figure S1. Box plots illustrating the DSC performance of the teams across the three lesions in DR lesion segmentation task. Ensemble represents the ensemble results of the top three algorithms.

Table S1. Quantitative results of top three teams in DR lesion segmentation task. Ensemble represents the ensemble results of the top three algorithms with a majority voting strategy.

Algorithm	IRMA				NPA				NV			
	DSC	SEN	PRE	SPE	DSC	SEN	PRE	SPE	DSC	SEN	PRE	SPE
A1	47.04	53.01	47.54	99.36	69.26	73.57	71.00	95.75	65.71	73.35	62.51	98.93
A2	48.32	57.56	47.07	99.19	67.36	74.72	69.20	94.47	65.70	77.07	60.33	98.68
A3	46.72	57.37	43.21	99.40	66.80	75.24	67.14	94.82	59.17	64.27	60.80	99.30
Ensemble	49.95	57.00	49.74	99.40	68.78	74.94	70.25	95.09	67.55	73.13	65.53	99.20

Table S2. Quantitative results of the top three algorithms in image quality assessment task. Ensemble represents the ensemble results of the top three algorithms with a majority voting strategy.

Algorithm	class	Sensitivity	Specificity	F1 score
	Poor	0.6842	0.9875	0.7536
B1	Good	0.6349	0.9627	0.6838
	Excellent	0.9763	0.7624	0.9536
	Poor	0.5789	0.9925	0.6984
B2	Good	0.7778	0.8853	0.6323
	Excellent	0.9169	0.8812	0.9392
	Poor	0.7368	0.9700	0.7179
B3	Good	0.4127	0.9733	0.5253
	Excellent	0.9822	0.6931	0.9471
	Poor	0.6842	0.9875	0.7536
Ensemble	Good	0.6349	0.9627	0.6838
	Excellent	0.9763	0.7624	0.9536

Table S3. Quantitative results of top three teams in DR grading task. Ensemble represents the ensemble results of the top three algorithms with a majority voting strategy.

Algorithm	class	Sensitivity	Specificity	F1 score
	non-DR	0.9631	0.9763	0.9721
C1	NPDR	0.8168	0.9373	0.8425
	PDR	0.7895	0.9425	0.6818
	non-DR	0.9724	0.9527	0.9679
C2	NPDR	0.7557	0.9608	0.8250
	PDR	0.8947	0.9310	0.7083
	non-DR	0.9770	0.9349	0.9636
C3	NPDR	0.8473	0.9176	0.8441
	PDR	0.5789	0.9741	0.6377
	non-DR	0.9724	0.9645	0.9724
Ensemble	NPDR	0.8473	0.9451	0.8672
	PDR	0.7895	0.9598	0.7317

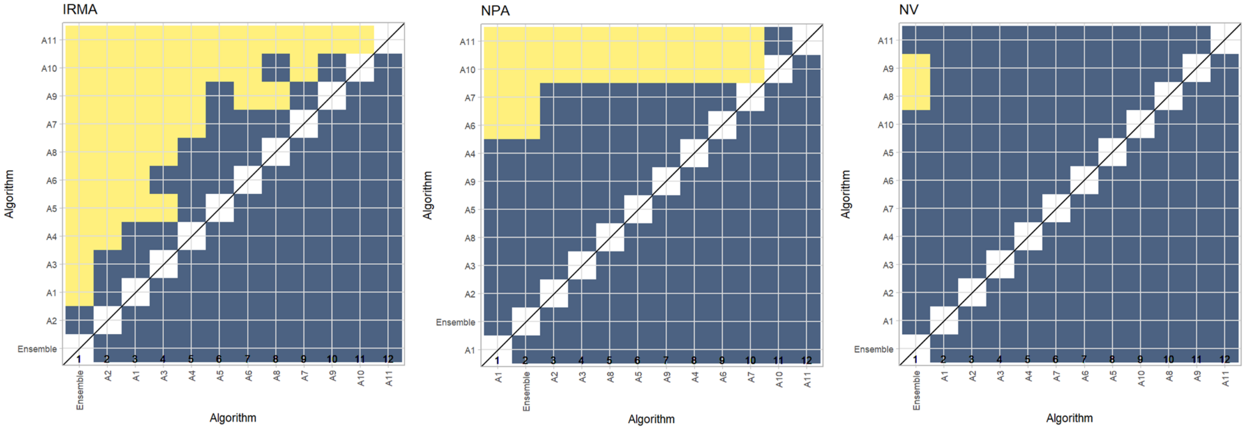

Figure S2. Significance maps depict incidence matrices of pairwise significant test results in DR lesion segmentation task for the one-sided Wilcoxon signed rank test at a 5% significance level with adjustment for multiple testing according to Holm. Yellow shading indicates that the mean DSC of the algorithm on the x-axis is significantly superior to those from the algorithm on the y-axis, and blue color indicates no significant difference.

Table S4. Summary of data augmentation techniques of top three algorithms in each task of the challenge. F, flipping; R, rotation; C, cropping; S, scaling; B, brightness; C, contrast; G, gamma; SP, sharpen; BL, blur; GD, grid distortion; CD, coarse dropout; CO, cut out; GN, gaussian noise; A, affine; MU, MixUp; CM, CutMix; TTA, test-time augmentation.

	Algorithm	Data augmentation										
		F	R	C	S	B	CT	G	SP	BL	TTA	Others
	A1	✓	✓		✓	✓	✓	✓	✓	✓	✓	A/GD/CD
Task 1	A2	✓	✓									
	A3	✓	✓		✓	✓	✓	✓	✓	✓		CO/GN
	B1	✓	✓		✓	✓	✓	✓	✓	✓	✓	
Task 2	B2	✓										
	B3	✓		✓	✓	✓	✓					MU/CM
	C1	✓	✓		✓	✓	✓	✓	✓	✓	✓	CD
Task 3	C2	✓										
	C3	✓	✓	✓								

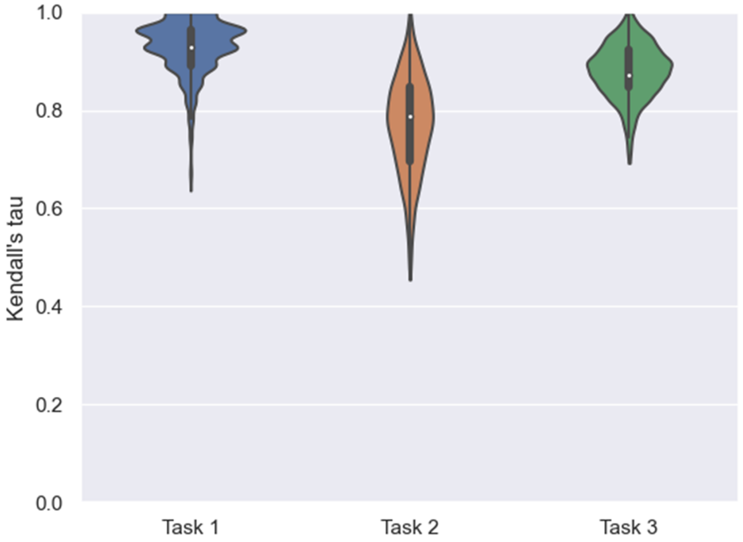

Figure S3. Violin plots of Kendall's τ for visualizing ranking stability based on bootstrapping. 1000 bootstrap samples are used for each task. The ranking list based on the full test data is compared pairwise with the ranking lists based on the individual bootstrap samples. Kendall's τ is computed for each pair of rankings, and a violin plot that simultaneously depicts a boxplot and a density plot is generated from the results.

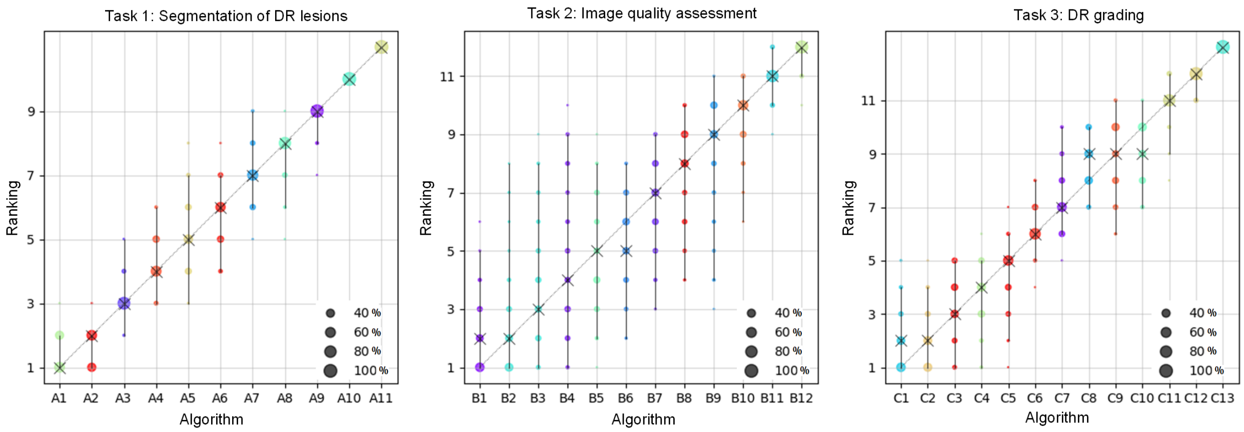

Figure S4. Blob plots for visualizing ranking stability based on bootstrap sampling. 1000 bootstrap samples are used for each task. The size of each circle is proportional to the relative frequency an algorithm obtained the corresponding rank across 1000 bootstrap samples. The median rank for each algorithm is indicated by a black cross. 95% bootstrap intervals across bootstrap samples are indicated by black lines.

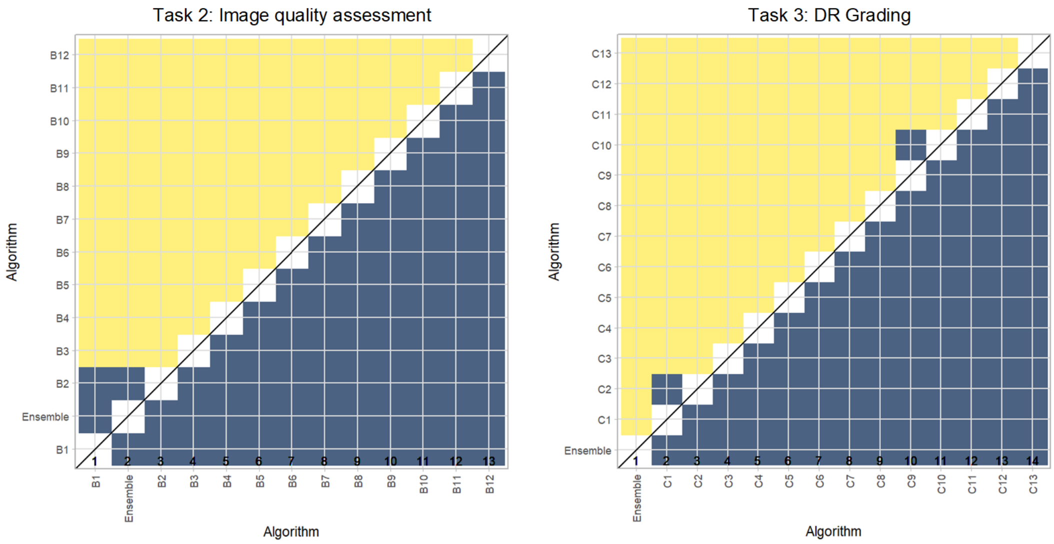

Figure S5. Significance maps depict incidence matrices of pairwise significant test results in the two classification tasks for the one-sided Wilcoxon signed rank test at a 5% significance level with adjustment for multiple testing according to Holm. Yellow shading indicates that the quadratic weighted kappa from the algorithm on the x-axis is significantly superior to those from the algorithm on the y-axis, and blue color indicates no significant difference.

References

1. Kwon, G., Kim, E., Kim, S., Bak, S., Kim, M., and Kim, J. (2023). Bag of Tricks for Developing Diabetic Retinopathy Analysis Framework to Overcome Data Scarcity. In Mitosis Domain Generalization and Diabetic Retinopathy Analysis Lecture Notes in Computer Science., B. Sheng and M. Aubreville, eds. (Springer Nature Switzerland), pp. 59–73. https://doi.org/10.1007/978-3-031-33658-4_7.
2. Qin, X., Zhang, Z., Huang, C., Dehghan, M., Zaiane, O.R., and Jagersand, M. (2020). U2-Net: Going deeper with nested U-structure for salient object detection. Pattern Recognition 106, 107404. https://doi.org/10.1016/j.patcog.2020.107404.
3. Lin, T.-Y., Goyal, P., Girshick, R., He, K., and Dollar, P. (2017). Focal Loss for Dense Object Detection. In Proceedings of the IEEE international conference on computer vision, pp. 2980-2988.
4. Kwon, G., and Kim, J. (2023). anears-vuno/DRAC22_MICCAI_FAI: v1.0.0 (v1.0.0). Zenodo. https://doi.org/10.5281/zenodo.10254200.
5. Choi, S., Jeoun, B., Anh, J., Jeong, J., Choi, Y., Kwon, D., Kim, U., and Shin, S. (2023). A Vision Transformer Based Deep Learning Architecture for Automatic Diagnosis of Diabetic Retinopathy in Optical Coherence Tomography Angiography. In Mitosis Domain Generalization and Diabetic Retinopathy Analysis Lecture Notes in Computer Science., B. Sheng and M. Aubreville, eds. (Springer Nature Switzerland), pp. 135–145. https://doi.org/10.1007/978-3-031-33658-4_13.
6. Liu, Z., Mao, H., Wu, C.-Y., Feichtenhofer, C., Darrell, T., and Xie, S. (2022). A convnet for the 2020s. In Proceedings of the IEEE/CVF conference on computer vision and pattern recognition, pp. 11976-11986.
7. Xie, E., Wang, W., Yu, Z., Anandkumar, A., Alvarez, J.M., and Luo, P. (2021). SegFormer: Simple and Efficient Design for Semantic Segmentation with Transformers. In Advances in Neural Information Processing Systems (Curran Associates, Inc.), pp. 12077–12090.
8. Liu, Z., Lin, Y., Cao, Y., Hu, H., Wei, Y., Zhang, Z., Lin, S., and Guo, B. (2021). Swin Transformer: Hierarchical Vision Transformer using Shifted Windows. In 2021 IEEE/CVF International Conference on Computer Vision (ICCV) (IEEE), pp. 9992–10002. https://doi.org/10.1109/ICCV48922.2021.00986.
9. K, Z. (1994). Contrast Limited Adaptive Histogram Equalization. Graphics Gems 0, pp. 474–485.
10. Loshchilov, I., and Hutter, F. (2019). Decoupled Weight Decay Regularization. Preprint at arXiv. https://doi.org/10.48550/arXiv.1711.05101.
11. Seoyoung, Jokulsarlon, and J-hyup. (2023). KT-biohealth/DRAC22: KT-biohealth/DRAC22_final (DRAC22). Zenodo. https://doi.org/10.5281/zenodo.10212156.
12. Krause, F., Heindl, D., Jebril, H., Karner, M., and Unterdechler, M. (2023). nnU-Net Pre- and Postprocessing Strategies for UW-OCTA Segmentation Tasks in Diabetic Retinopathy Analysis. In Mitosis Domain Generalization and Diabetic Retinopathy Analysis Lecture Notes in Computer Science., B. Sheng and M. Aubreville, eds. (Springer Nature Switzerland), pp. 5–15. https://doi.org/10.1007/978-3-031-33658-4_1.
13. Isensee, F., Jaeger, P.F., Kohl, S.A.A., Petersen, J., and Maier-Hein, K.H. (2021). nnU-Net: a self-configuring method for deep learning-based biomedical image segmentation. Nat Methods 18, 203–211. https://doi.org/10.1038/s41592-020-01008-z.
14. Krause, F. (2023). flixmk/DRAC22-JKU: DRAC22-JKU (v0.1.0). Zenodo. https://doi.org/10.5281/zenodo.10254707.
15. Tan, M., and Le, Q. (2019). EfficientNet: Rethinking Model Scaling for Convolutional Neural Networks. In Proceedings of the 36th International Conference on Machine Learning (PMLR), pp. 6105–6114.
16. Bao, H., Dong, L., Piao, S., and Wei, F. (2022). BEiT: BERT Pre-Training of Image Transformers. Preprint at arXiv. https://doi.org/10.48550/arXiv.2106.08254.
17. Brock, A., De, S., Smith, S.L., and Simonyan, K. (2021). High-Performance Large-Scale Image Recognition Without Normalization. In Proceedings of the 38th International Conference on Machine Learning (PMLR), pp. 1059–1071.
18. Hou, J., Xiao, F., Xu, J., Zhang, Y., Zou, H., and Feng, R. (2023). Deep-OCTA: Ensemble Deep Learning Approaches for Diabetic Retinopathy Analysis on OCTA Images. In Mitosis Domain Generalization and Diabetic Retinopathy Analysis Lecture Notes in Computer Science., B. Sheng and M. Aubreville, eds. (Springer Nature Switzerland), pp. 74–87. https://doi.org/10.1007/978-3-031-33658-4_8.
19. Szegedy, C., Vanhoucke, V., Ioffe, S., Shlens, J., and Wojna, Z. (2016). Rethinking the Inception Architecture for Computer Vision. In Proceedings of the IEEE conference on computer vision and pattern recognition, pp. 2818–2826.

20. Xie, S., Girshick, R., Dollar, P., Tu, Z., and He, K. (2017). Aggregated Residual Transformations for Deep Neural Networks. In 2017 IEEE Conference on Computer Vision and Pattern Recognition (CVPR) (IEEE), pp. 5987–5995. https://doi.org/10.1109/CVPR.2017.634.

21. Dosovitskiy, A., Beyer, L., Kolesnikov, A., Weissenborn, D., Zhai, X., Unterthiner, T., Dehghani, M., Minderer, M., Heigold, G., Gelly, S., et al. (2021). An Image is Worth 16x16 Words: Transformers for Image Recognition at Scale. Preprint at arXiv. https://doi.org/10.48550/arXiv.2010.11929.

22. Wang, Y., Shen, Y., Yuan, M., Xu, J., Yang, B., Liu, C., Cai, W., Cheng, W., and Wang, W. (2021). A Deep Learning-based Quality Assessment and Segmentation System with a Large-scale Benchmark Dataset for Optical Coherence Tomographic Angiography Image. Preprint at arXiv. https://doi.org/10.48550/arXiv.2107.10476.

23. Zhang, H., Cisse, M., Dauphin, Y.N., and Lopez-Paz, D. (2018). mixup: Beyond Empirical Risk Minimization. Preprint at arXiv. https://doi.org/10.48550/arXiv.1710.09412.

24. Yun, S., Han, D., Chun, S., Oh, S.J., Yoo, Y., and Choe, J. (2019). CutMix: Regularization Strategy to Train Strong Classifiers With Localizable Features. In 2019 IEEE/CVF International Conference on Computer Vision (ICCV) (IEEE), pp. 6022–6031. https://doi.org/10.1109/ICCV.2019.00612.

25. Hou, J., and Xiao. (2023). FDU-VTS/DRAC: Code Release 1.0.0 (v1.0.0). Zenodo. https://doi.org/10.5281/zenodo.10210181.

26. Li, Y., Zeghlache, R., Brahim, I., Xu, H., Tan, Y., Conze, P.-H., Lamard, M., Quellec, G., and El Habib Daho, M. (2023). Segmentation, Classification, and Quality Assessment of UW-OCTA Images for the Diagnosis of Diabetic Retinopathy. In Mitosis Domain Generalization and Diabetic Retinopathy Analysis Lecture Notes in Computer Science., B. Sheng and M. Aubreville, eds. (Springer Nature Switzerland), pp. 146–160. https://doi.org/10.1007/978-3-031-33658-4_14.

27. Huang, G., Liu, Z., Van Der Maaten, L., and Weinberger, K.Q. (2017). Densely Connected Convolutional Networks. In 2017 IEEE Conference on Computer Vision and Pattern Recognition (CVPR) (IEEE), pp. 2261–2269. https://doi.org/10.1109/CVPR.2017.243.

28. LI, Y., and EL HABIB DAHO, M. (2023). Mostafa-EHD/Diabetic_Retinopathy_OCTA: important release (v1.0). Zenodo. https://doi.org/10.5281/zenodo.10209637.
