# Supplementary material for: DRAC 2022: A public benchmark for diabetic retinopathy analysis on ultra-wide optical coherence tomography angiography images
Source: Patterns (N Y). 2024 Feb 8;5(3):100929. doi: 10.1016/j.patter.2024.100929 (PMC10935505; doi:10.1016/j.patter.2024.100929)
Supplement: Document S2. Article plus supplemental information [file mmc2.pdf]

Descriptor

Patterns

DRAC 2022: A public benchmark for diabetic retinopathy analysis on ultra-wide optical coherence tomography angiography images

Graphical abstract

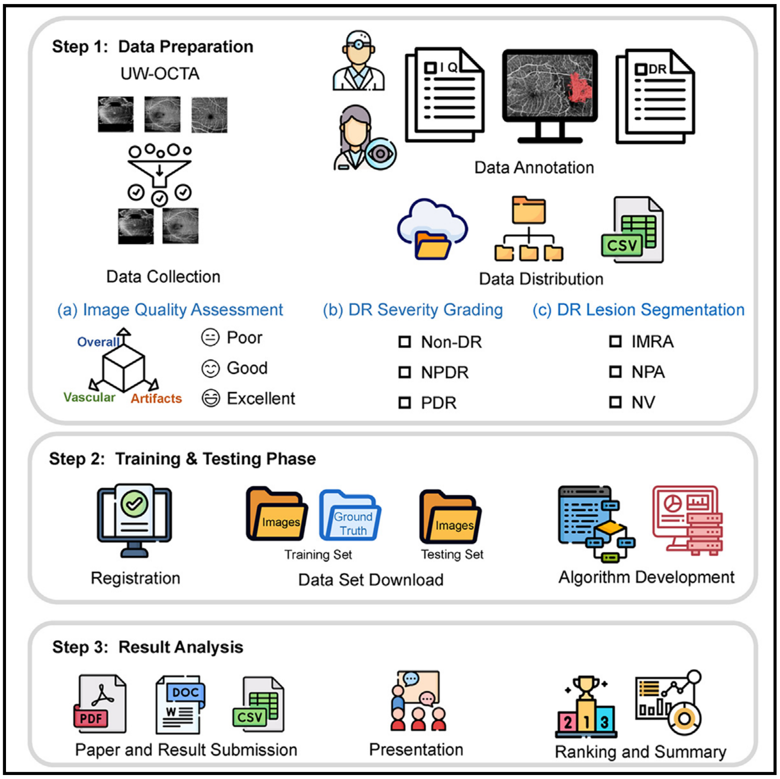

Highlights

- Provides the DRAC dataset, top-performing methods, and results
- Presents the deep learning methods in DR grading and lesion segmentation
- Summarizes the strategies for improving the model performance

Authors

Bo Qian, Hao Chen,
Xiangning Wang, ..., Yih Chung Tham,
Tien Yin Wong, Bin Sheng

Correspondence

huarting99@sjtu.edu.cn (H.L.),
thamyc@nus.edu.sg (Y.C.T.),
wongtienyin@tsinghua.edu.cn (T.Y.W.),
shengbin@sjtu.edu.cn (B.S.)

In brief

The DRAC challenge explored the use of artificial intelligence to tackle clinical tasks related to diabetic retinopathy (DR) using ultra-wide OCTA imaging. Here, the organizers present a comprehensive summary of the top three algorithms and the results for each task, including image quality assessment, lesion segmentation, and DR grading. These methods provide new insights into the diagnosis of DR and could potentially enhance the diagnostic capabilities of healthcare professionals in DR evaluation.

Qian et al., 2024, Patterns *5*, 100929
March 8, 2024 © 2024 The Author(s).
https://doi.org/10.1016/j.patter.2024.100929

CellPress

Patterns

CellPress
OPEN ACCESS

Descriptor

DRAC 2022: A public benchmark for diabetic retinopathy analysis on ultra-wide optical coherence tomography angiography images

Bo Qian,[1,2,24] Hao Chen,[3,4,24] Xiangning Wang,[1,5,24] Zhouyu Guan,[1,24] Tingyao Li,[1,2,24] Yixiao Jin,[6] Yilan Wu,[6] Yang Wen,[7] Haoxuan Che,[3] Gitaek Kwon,[8] Jaeyoung Kim,[8] Sungjin Choi,[9] Seoyoung Shin,[9] Felix Krause,[10] Markus Unterdechler,[10] Junlin Hou,[11] Rui Feng,[11,12] Yihao Li,[13,14] Mostafa El Habib Daho,[13,14] Dawei Yang,[15] Qiang Wu,[5] Ping Zhang,[16,17,18]

(Author list continued on next page)

[1]Shanghai Belt and Road International Joint Laboratory for Intelligent Prevention and Treatment of Metabolic Disorders, Department of Computer Science and Engineering, School of Electronic, Information, and Electrical Engineering, Shanghai Jiao Tong University, Department of Endocrinology and Metabolism, Shanghai Sixth People's Hospital Affiliated to Shanghai Jiao Tong University School of Medicine, Shanghai Diabetes Institute, Shanghai Clinical Center for Diabetes, Shanghai 200240, China
[2]MOE Key Laboratory of AI, School of Electronic, Information, and Electrical Engineering, Shanghai Jiao Tong University, Shanghai 200240, China
[3]Department of Computer Science and Engineering, The Hong Kong University of Science and Technology, Hong Kong 999077, China

(Affiliations continued on next page)

THE BIGGER PICTURE Diabetic retinopathy (DR) is a common eye disease that can lead to visual impairment and even blindness. The study of DR is an important area that significantly affects the lives of millions of people worldwide. Understanding and managing DR is not only a medical challenge but also a societal one, emphasizing the need for early detection and intervention. A key to such understanding is ultra-wide optical coherence tomography angiography (UW-OCTA), a non-invasive imaging modality that could enable precise assessment of microvascular changes in retinal layers. To this end, we organized a medical image challenge and provided a UW-OCTA dataset for developing the computer-aided diagnostic system for DR diagnosis. The dataset can potentially accelerate the development of advanced artificial intelligence technologies and ultimately improve patient care.

SUMMARY

We described a challenge named "DRAC - Diabetic Retinopathy Analysis Challenge" in conjunction with the 25th International Conference on Medical Image Computing and Computer Assisted Intervention (MICCAI 2022). Within this challenge, we provided the DRAC datset, an ultra-wide optical coherence tomography angiography (UW-OCTA) dataset (1,103 images), addressing three primary clinical tasks: diabetic retinopathy (DR) lesion segmentation, image quality assessment, and DR grading. The scientific community responded positively to the challenge, with 11, 12, and 13 teams submitting different solutions for these three tasks, respectively. This paper presents a concise summary and analysis of the top-performing solutions and results across all challenge tasks. These solutions could provide practical guidance for developing accurate classification and segmentation models for image quality assessment and DR diagnosis using UW-OCTA images, potentially improving the diagnostic capabilities of healthcare professionals. The dataset has been released to support the development of computer-aided diagnostic systems for DR evaluation.

INTRODUCTION

Diabetic retinopathy (DR) is one of the most common complications caused by diabetes.[1] Patients with DR are more likely to get vision impairment and even blindness than healthy individuals. DR affects a large amount of the working-age population worldwide. According to the International Diabetes Federation,[2] it is estimated that about 700 million people in the world are

This is an open access article under the CC BY license (http://creativecommons.org/licenses/by/4.0/).

Xiaokang Yang,[2] Yiyu Cai,[19] Gavin Siew Wei Tan,[20] Carol Y. Cheung,[15] Weiping Jia,[1] Huating Li,[1,*]
Yih Chung Tham,[20,21,22,*] Tien Yin Wong,[6,20,23,*] and Bin Sheng[1,2,25,*]

[4]Department of Chemical and Biological Engineering, The Hong Kong University of Science and Technology, Hong Kong 999077, China
[5]Shanghai Sixth People's Hospital Affiliated to Shanghai Jiao Tong University School of Medicine, Shanghai 200233, China
[6]Tsinghua Medicine, Tsinghua University, Beijing 100084, China
[7]School of Electronic and Information Engineering, Shenzhen University, Shenzhen 518060, China
[8]VUNO, Inc., Seoul 06536, Korea
[9]AI/DX Convergence Business Group, KT, Seongnam 13606, Korea
[10]Johannes Kepler University Linz, Linz 4040, Austria
[11]School of Computer Science, Shanghai Key Laboratory of Intelligent Information Processing, Fudan University, Shanghai 200433, China
[12]Academy for Engineering and Technology, Fudan University, Shanghai 200433, China
[13]LaTIM UMR 1101, INSERM, 29609 Brest, France
[14]University of Western Brittany, 29238 Brest, France
[15]Department of Ophthalmology and Visual Sciences, The Chinese University of Hong Kong, Hong Kong 999077, China
[16]Department of Computer Science and Engineering, The Ohio State University, Columbus, OH 43210, USA
[17]Department of Biomedical Informatics, The Ohio State University, Columbus, OH 43210, USA
[18]Translational Data Analytics Institute, The Ohio State University, Columbus, OH 43210, USA
[19]School of Mechanical and Aerospace Engineering, Nanyang Technological University, Singapore 639798, Singapore
[20]Singapore Eye Research Institute, Singapore National Eye Centre, Singapore 168751, Singapore
[21]Centre for Innovation and Precision Eye Health; and Department of Ophthalmology, Yong Loo Lin School of Medicine, National University of Singapore, Singapore 119228, Singapore
[22]Ophthalmology and Visual Sciences Academic Clinical Program, Duke-NUS Medical School, Singapore 169857, Singapore
[23]School of Clinical Medicine, Beijing Tsinghua Changgung Hospital, Beijing 102218, China
[24]These authors contributed equally
[25]Lead contact
*Correspondence: huarting99@sjtu.edu.cn (H.L.), thamyc@nus.edu.sg (Y.C.T.), wongtienyin@tsinghua.edu.cn (T.Y.W.), shengbin@sjtu.edu.cn (B.S.)
https://doi.org/10.1016/j.patter.2024.100929

expected to have diabetes by 2045, and one-third of them will have DR. DR is diagnosed by visually inspecting retinal fundus images for the presence of retinal lesions, such as exudates, microaneurysm (MA), intraretinal microvascular abnormality (IRMA), and neovascularization (NV).[3] Hence, the detection of these lesions is significant for DR diagnosis.

Regular DR screening and timely treatment can be implemented to reduce the risks of vision loss and blindness.[4,5] However, there are many challenges to population screening. First, comprehensive DR screening puts a heavy burden on ophthalmologists. Especially in developing countries and rural parts, there may not be enough medical resources and ophthalmologists to perform the DR screening.[6–9] Second, DR screening relies heavily on the experience of ophthalmologists. Differences in the experience of professional ophthalmologists may lead to different diagnoses, and the inadequate training of ophthalmologists can also result in misdiagnosis and low accuracy in DR screening.[8,10] Third, systematic DR screening is associated with complicated social management and economic burden. Hence, the implementation of an efficient computer-aided system becomes indispensable in supporting manual DR screening. Such a system can assist in achieving precise diagnoses, thereby significantly alleviating the workload of ophthalmologists.[11–13]

The most commonly used imaging modalities for the clinical diagnosis of DR include fundus photography, fluorescein angiography (FA), and optical coherence tomography angiography (OCTA). Fundus photography is a common modality for rapid screening of DR. It effectively captures the distribution of hard exudates and retinal changes in severe non-proliferative DR (NPDR). However, it is difficult to detect early or small neovascular lesions. FA primarily detects the presence of NV but involves invasive fundus imaging and is unsuitable for patients with allergies, pregnancy, or poor kidney function. OCTA provides a non-invasive means of identifying changes in NV and assists ophthalmologists in diagnosing proliferative DR (PDR). Further, ultra-wide OCTA (UW-OCTA) reveals a broader peripheral retinal area beyond the scope of typical OCTA. For example, UW-OCTA imaging allows the assessment of peripheral retinal vascular networks, the detection of early stages of NPDR through capillary flow analysis, and the localization of NV lesions in patients with PDR.[14,15] Several studies have used UW-OCTA images for DR diagnosis, screening, and follow-up purposes.[16–18]

In particular, Pichi et al.[18] conducted a comparative analysis of UW-OCTA against UW-field FA (UWF-FA) and UWF color fundus photography (UWF-CP) for detecting NV in eyes with PDR. Their findings indicated that WF-OCTA can identify NV that is not evident in UWF-CP and serves as a swifter and safer alternative to UWF-FA for PDR monitoring, delivering comparable diagnostic accuracy. Khalid et al.[16] conducted a retrospective observational case series comprising patients clinically diagnosed with PDR or severe NPDR. They reported that 12 × 12 mm UW-OCTA imaging exhibits superior PDR detection rates compared to clinical examination. This implies the non-invasive potential of this modality for early NV detection and characterization. Moreover, Kim et al.[19] quantified the foveal avascular zone (FAZ), vessel density (VD), and NPA across three distinct OCTA field sizes: 3 × 3, 6 × 6, and 10 × 10 mm. Their experiment revealed that NPA measurements from the larger 10 × 10 mm scan were the sole discriminating parameter for the three NPDR stages, with the 10 × 10 mm scan demonstrating the highest sensitivity in determining five-grade DR severity.

Table 1. Detailed descriptions for each image quality level

Image quality level	Overall quality	Artifacts	Vascular quality
Poor	insufficient	severe	blurring
Good	moderate blurring or stripe noise	moderate	moderate blurring
Excellent	slight blurring or with slight stripe noise	slight	clear or slight blurring

Similarly, Zhu et al.[20] performed a prospective study comparing NV detection among four different OCTA field sizes: 3 × 3 mm angiography, 6 × 6 mm angiography, 15 × 9 mm montage, and 12 × 12 mm angiography. Both the 12 × 12 mm angiography scan and the 15 × 9 mm montage scanning exhibited high detection rates, but the former offered the advantage of taking less time to perform. All of these studies collectively underscore the potential of UW-OCTA imaging in DR detection.

Artificial intelligence challenges play a crucial role in advancing the application of deep learning techniques in medical image analysis. These challenges define one or more clinically significant tasks and provide the corresponding datasets, encouraging participants to develop algorithms for these tasks and enabling a fair comparison. Many challenges have been organized for DR analysis, such as ROC,[21] IDRiD,[22] and DeepDRiD,[23] all employing fundus photography as the imaging modality. These initiatives have led to the creation of a multitude of state-of-the-art (SOTA) algorithms, significantly contributing to the research community. However, to our knowledge, there is a dearth of publicly available UW-OCTA datasets for evaluating DR. Against this background, we organized the Diabetic Retinopathy Analysis Challenge (DRAC) at the 25th International Conference on Medical Image Computing and Computer Assisted Intervention (MICCAI 2022), with the aim of establishing a benchmark and evaluation framework for the automated analysis of DR using UW-OCTA images.

In this paper, we describe in detail the DRAC dataset, challenge setup, and the top-performing solutions. We also report and analyze the challenge results, including ranking stability, model ensemble, and statistical significance. Finally, we discuss the clinical value of the dataset for future users, strategies to improve the model performance, limitations of the study, and the future work.

Methods

Data

All the images in the DRAC dataset were acquired with the VG200D UW swept-source OCTA device. The scan captures a 12 × 12 mm area of the inner retinal layer, centered on the fovea. A total of 1,103 UW-OCTA images were collected with a resolution of 1,024 × 1,024 pixels. There are three types of annotations for corresponding clinically relevant tasks. First, image quality has a profound impact on disease diagnosis, and high-quality images are essential for accurate DR diagnosis. Therefore, the first task is image quality assessment, including poor, good, and excellent quality levels. The images of good and excellent quality can then be used for two other DR-related tasks: DR grading and DR lesion segmentation. One of the advantages of UW-OCTA imaging is its ability to detect NV, which is a critical indicator of PDR. Therefore, the second task is to identify PDR images from non-DR and NPDR images. In the lesion segmentation task, there are three different lesions to be segmented: IRMA, non-perfusion area (NPA), and NV. These three lesions are important morphological features of DR severity and can help to visualize pathological features of DR.[24,25]

In the process of image annotating, many factors affect the image quality,[26,27] such as artifacts, vascular quality, etc. The specific annotation standard of image quality assessment is shown in Table 1. For DR grading, the fundus photograph corresponding to the UW-OCTA image was used to grade the DR, with the specific grading standard for non-DR, NPDR, and PDR referring to the international clinical DR severity scale.[28] For each task, two ophthalmologists participated in the annotation process. First, the two ophthalmologists independently annotated the labels for each image according to the annotation standards. In the event of any disagreement, two additional, more experienced ophthalmologists were involved to help reach a consensus on the annotation.

The data division method for each task in this challenge is as follows. For image quality assessment task, the images were split into 60% for training (665 images) and 40% for testing (438 images). For the DR-grading task and the DR lesion segmentation task, the data division method was the same as the task of image quality assessment, but in the DR-grading task, we removed the images with the poor quality levels and images that caused considerable controversies about the DR grade by ophthalmologists, and then the rest of the images were retained as the training set (611 images) and the test set (386 images), respectively. In the DR lesion segmentation task, we only retained images that show representative lesions to form the training set (109 images) and the test set (65 images). The images were stored in gray *png* format. The ground truth of training set for the classification task was stored and provided in a CSV file. For the segmentation task, the ground truth was provided in the form of binary masks stored in *png* format.

Challenge setup

The DRAC challenge aimed at providing a benchmark for evaluating the algorithms that are used for the automatic DR analysis using UW-OCTA images. It addressed the current lack of publicly available UW-OCTA datasets for fair performance evaluation of DR diseases. The challenge was subdivided into three tasks as follows. Image quality assessment and DR grading are both three-class classification tasks, so we have grouped them together as task 2 and task 3 in this challenge. Figure 1 shows the example images in each task.

(1) Task 1: segmentation of DR lesions. There are three types of lesions to be segmented, including IRMA, NPA, and NV.
(2) Task 2: image quality assessment. Classification of the image quality levels, including poor quality level, good quality level, and excellent quality level.
(3) Task 3: DR grading. Classification of DR grades according to the severity level of DR, including non-DR, NPDR, and PDR.

The organization of the challenge referred to the Biomedical Image Analysis Challenges guideline.[29] The challenge was officially announced at MICCAI 2022 and was hosted on the Grand

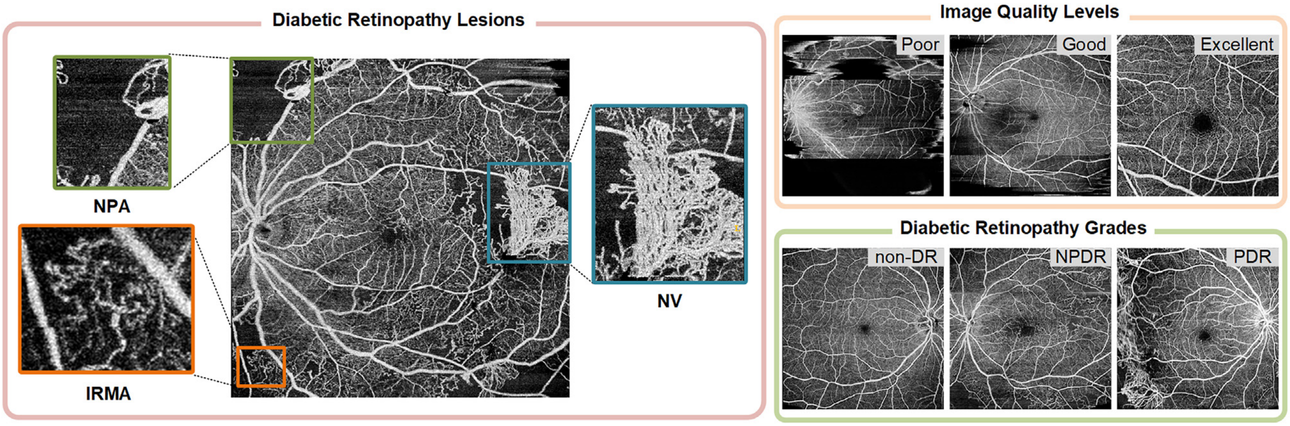

Figure 1. Examples of UW-OCTA images in three tasks of the challenge
IRMA, intraretinal microvascular abnormality; NPA, nonperfusion area; NV, neovascularization; non-DR, non-diabetic retinopathy; NPDR, non-proliferative diabetic retinopathy; PDR, proliferative diabetic retinopathy.

Challenge platform. The challenge website is available at https://drac22.grand-challenge.org. On the challenge website, the participants could have access to the dataset after they registered on the website and signed the challenge rule agreement. In addition, participants could browse the challenge rules and news, submit results, and find their rankings on the challenge website. In the case of multiple submissions, only the most recent run was counted for the final challenge result. We also provided submission guidelines for the participants. The challenge was launched in July 2022 by releasing the training dataset. The test set was released on August 8th, 2022, and the challenge submission was opened between August 8th, 2022, and September 12th, 2022. Each participating team was required to submit a method description paper with a minimum of 4 pages before October 8th, 2022. When participating in multiple tasks, each team could either submit several papers or a single paper reporting all methods. The details of the evaluation method can be seen in the supplemental information: evaluation metrics.

Finally, a total of 91 teams and individuals from more than 25 different countries or regions signed the challenge rule agreement consent form and downloaded the dataset throughout the challenge. Out of them, 17 teams submitted a total of 18 method description papers before the deadline, where 11, 12, and 13 different methods were reported for the three tasks, respectively. Some teams participated in two or more tasks and chose to report their methods in one single paper. During the satellite event at MICCAI 2022 on September 18th, 2022, we summarized the challenge results and invited the top-ranked teams to present their algorithms. The summary of the top three algorithms for each task is shown in Note S1. For a more in-depth description of a particular approach, please refer to MICCAI Challenge Proceedings.[30]

RESULTS

The final rankings of the three tasks are shown in Figure 2. To facilitate clarity, we adopt the labels A, B, and C to denote the algorithms corresponding to task 1 (DR lesion segmentation), task 2 (image quality assessment), and task 3 (DR grading), respectively, followed by a number indicating the ranking of the algorithm in this task. For example, A1, B1, and C1 represent the first-ranked algorithms in task 1, task 2, and task 3, respectively. We first present the results obtained by the participating teams and then analyze the ranking stability of these algorithms. We also present the ensemble of the results of the top three algorithms in each task and report the statistical significance analysis of the algorithms.

Task 1: Segmentation of DR lesions

In the task of DR lesion segmentation, out of the total 24 teams that submitted the results on the test set, 11 teams submitted method description papers. The average dice similarity coefficient (DSC) of the top ten teams ranged from 40.95% to 60.67%. The DSC distribution of each class of lesion is shown in Figure S1. Among these three lesions, IRMA has the lowest segmentation performance, with DSCs ranging from 29.53% to 47.04% for the top ten algorithms. NPA has the highest segmentation performance, with DSCs ranging from 46.59% to 69.26% for the top ten algorithms, and NV ranks second, with DSCs ranging from 46.73% to 65.71% for the top ten algorithms. The complex lesion features could explain the low segmentation performance of IRMA because IRMAs are usually thin vessels that spread throughout the image. In addition to the DSC used in the challenge, we also use sensitivity (SEN), precision (PRE) and specificity (SPE) to evaluate the segmentation performance of each method. The quantitative results of the top three methods are shown in Table S1.

Task 2: Image quality assessment

In the task of image quality assessment, a total of 45 teams submitted results, and 12 teams submitted method description papers, where the quadratic weighted kappa (QWK) of the top ten teams ranged from 0.7246 to 0.8090. Table S2 shows the quantitative results of the top three algorithms for the metrics of sensitivity, specificity, and F1 score. Combined with the confusion matrices of the top three methods in Figure 3, there are two notable observations. Firstly, there is a tendency to misclassify some images as having excellent quality regardless of whether

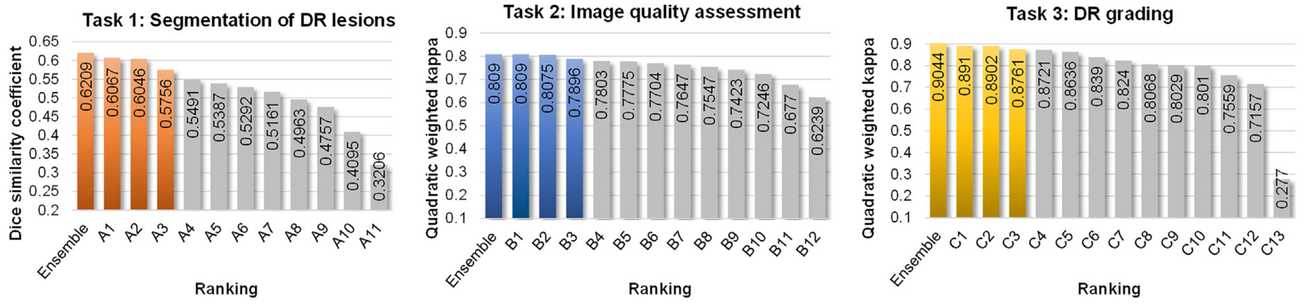

Figure 2. Bar charts of the final rankings for three tasks
The colored bars show the ensemble and the top three scores in each task. Ensemble represents the ensemble results of the top three algorithms.

their actual quality is good or poor. This phenomenon can be attributed to the data imbalance of the dataset, where 80% of the images have an excellent quality level, while the remaining 20% have poor and good quality levels. This imbalance tends to bias the training toward the majority class, as the models strive to minimize the loss function. Although techniques like resampling can improve the classification performance of the minority class, they cannot fully compensate for the performance gap between the minority and majority classes caused by the data imbalance. Secondly, another noticeable trend is the misclassification of images between the adjacent classes, where some images with a poor quality level are misclassified as good quality level while some images with a good quality level are misclassified as excellent quality level. This can be explained by subtle feature distinctions between adjacent image quality levels, making it difficult for the network to classify correctly. Combined with the data imbalance issue, we observe that, for example, in ensemble results, 30% of the images with good image quality are misclassified as excellent image quality.

Task 3: DR grading

In the task of DR grading, a total of 45 teams submitted results, and 13 teams submitted method description papers, where the quadratic weighted kappa of the top ten teams ranged from 0.7157 to 0.8910. Table S3 shows the quantitative results of the top three algorithms for the metrics of sensitivity, specificity, and F1 score. Combined with the confusion matrices of the top three methods in Figure 3, we can see that the non-DR class achieves the highest accuracy, while the misclassifications mainly occur between the NPDR and PDR classes. Apart from the influence of the data imbalance, where the non-DR, NPDR, and PDR account for approximately 55%, 35%, and 10% of the dataset images, respectively, another possible factor is that the NV regions, which are a representative indicator of PDR, are usually small in size, posing a challenge for the classification network to accurately detect these areas, thereby leading to misclassifications between NPDR and PDR.

Ranking stability

Inspired by the challengeR toolkit,[31] we performed bootstrapping (1,000 bootstrap samples) to assess the stability of rankings with respect to sampling variability. To quantitatively assess the ranking stability, the agreement of the challenge ranking and the ranking of each bootstrap on the test set was determined via Kendall's τ, which is a rank correlation coefficient with a value between -1 (reverse ranking order) and 1 (identical ranking order). The violin plots shown in Figure S3 illustrate the bootstrap results for each task. We obtained Kendall's τ of 0.9313, 0.7697, and 0.8802 for the tasks of DR lesion segmentation, image quality assessment, and DR grading, respectively. Figure S4 shows a blob plot of the bootstrap rankings for each task.

Ensemble of top three algorithms

The ensemble of networks has shown great power in improving model performance.[32–34] Thus, it is interesting to explore the ensemble of the top three methods in each task. For the segmentation task, we use three forms of ensemble to generate the final segmentation output, including logical AND, logical OR, and majority voting. The DSCs of the three forms of ensemble results are 47.10%, 45.36%, and 49.95% for IRMA, 67.91%, 66.83%, and 68.78% for NPA, and 63.33%, 60.23%, and 67.55% for NV. For each of the three classes, the highest DSC is achieved by the majority voting strategy, of which the averaged DSC is 62.09%—which is 1.42% higher than the best result of the participating algorithms. The detailed performance of the ensemble result with a majority voting is shown in Table S1. For the two classification tasks, we use a majority voting strategy from the top three results to generate the ensemble result. For image quality assessment, the quadratic weighted kappa of the ensemble result is 0.8090, which is equal to the best score of participating algorithms. The detailed performance of the ensemble result is shown in Table S2. For DR grading, the quadratic weighted kappa of the ensemble result is 0.9044, which is 1.4% higher than the best score of participating algorithms. The detailed performance of the ensemble result is shown in Table S3. From the ensemble results in both the segmentation task and the classification task, we can see that the ensemble of the model has great power in improving the performance of deep learning methods.

Statistical significance analysis of the algorithms

For each task of the challenge, the statistical comparison of the score of each team is done with the one-tailed Wilcoxon signed rank test at a 5% significance level. The challengeR toolkit[31] is used to perform the significance analysis and generate the significance map. For the task of DR lesion segmentation, the significance maps in Figure S2 show the results of the statistical significance analysis for the three lesions. In conclusion, there

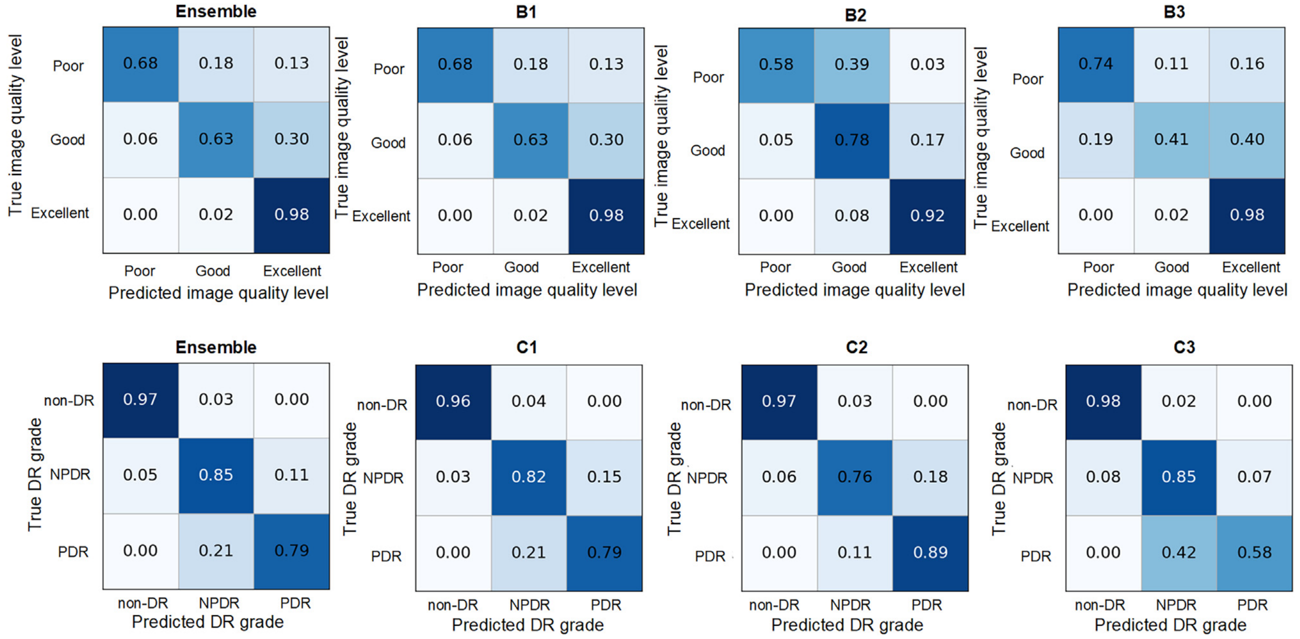

Figure 3. Confusion matrix for image quality assessment task and DR-grading task

The top row is the image quality assessment task and the bottom row is the DR-grading task. From left to right are the ensemble and first-, second-, and third-ranked results, respectively. Ensemble represents the ensemble results of the top three algorithms.

is a significant difference between multiple algorithm pairs for IRMA segmentation, but for the segmentation of NPA and NV, only a few pairs of algorithms show significant differences. For the two classification tasks, the statistical comparison of the score of each team is shown in Figure S5. In the task of image quality assessment, there are no significant differences among B1, the ensemble model, and B2. In the task of DR grading, the ensemble model is significantly superior to the algorithms C1, C2, and C3, but there is no significant difference between C1 and C2.

DISCUSSION

Clinical value of the dataset for future users

UW-OCTA imaging offers significant advantages in the monitoring and management of DR. First, compared to OCTA images, UW-OCTA can provide a wider field of view, allowing ophthalmologists to detect more abnormal lesions and microvascular changes. Second, compared to fundus photography and FA, OCTA can provide more comprehensive and detailed information about the retinal vascular structure in a non-invasive manner. OCTA does not require the use of contrast agents, which is an important advantage for patients who are allergic or intolerant to contrast agents. In contrast, FA involves injecting contrast agents into the patient's body, which can lead to allergic reactions or other discomfort. Moreover, OCTA can capture tiny blood vessels, allowing ophthalmologists to detect microvascular lesions and microvascular occlusions earlier, facilitating timely intervention.

We have released the largest UW-OCTA dataset (DRAC dataset) with corresponding annotations for three DR-related tasks: image quality assessment, DR lesion segmentation, and DR grading. Firstly, the image quality assessment task helps ensure the reliability of retinal images obtained from OCTA devices. This is crucial for accurate disease diagnosis, as diagnoses based on poor-quality images can lead to erroneous judgments. Through this task, we can raise the standards of medical image acquisition and ensure that healthcare professionals and researchers have high-quality data for further analysis and diagnosis. Secondly, the DR lesion segmentation task provides automated segmentation tools for different types of DR lesions, including IRMA, NPA, and NV. This means that ophthalmologists and researchers can identify lesion areas more quickly and accurately, enabling earlier intervention and treatment. This is vital for reducing the risk of blindness and improving the quality of life for patients. Finally, the DR-grading task helps to classify the severity of DR in patients, with particular emphasis on the detection of PDR, given the high detection rate achieved using OCTA images. This will help ophthalmologists to develop more accurate treatment plans and monitor the progression. We believe that this dataset can provide valuable resources for the research community and pave the way for the development of artificial intelligence algorithms.

Strategies to improve the model performance

We summarize the characteristics of the top three competing solutions, as shown in Tables 2 and S4, and then recognize a selection of frequently employed strategies with the potential to improve algorithmic performance.

For the data preprocessing and data augmentation, when considering input image resolution, most teams choose to maintain the original image size to prevent any loss of features. Regarding image normalization, the leading teams often opt for scaling pixel values within the 0–1 range or implementing zero-mean unit variance normalization. Such normalization of

Table 2. Summary of model architecture, loss function, and optimizer from top three algorithms in each task of the challenge

	Algorithm	Model architecture	Loss function	Optimizer	Score
Task 1	A1	U2Net	WDL + FL/CE	AdamW	0.6067
	A2	ConvNext + SegFormer + Swin Transformer	DL + FL/CE	AdamW	0.6046
	A3	nnUNet	DL + CE	SGD	0.5756
Task 2	B1	EfficientNet	Smooth L1	AdamW	0.8090
	B2	BEIT + NFNet	CE	AdamW	0.8075
	B3	Inception-V3 + SE-ResNeXt + ViT	CE	SGD	0.7896
Task 3	C1	EfficientNet	Smooth L1	AdamW	0.8910
	C2	BEIT	CE	AdamW	0.8902
	C3	DenseNet121 + Efficientnet	CE	Adam	0.8761

In the score column, task 1 utilizes the DSC score, while tasks 2 and 3 employ QWK scores. DL, dice loss; WDL, weighted dice loss; FL, focal loss; CE: cross-entropy; SGD, stochastic gradient descent.

input image pixel values can help accelerate the convergence speed of the model, mitigating issues like vanishing and exploding gradients to enhance training efficiency. Additionally, it can maintain stable numerical computations and enhance overall model performance. All teams used some form of data augmentation to enhance their models. The most popular image transformation is flipping, which was adopted by all top three teams. Other commonly used image transformations include rotation, scaling, brightness modification, contrast modification, etc. In addition, more complex multi-image data augmentation techniques are also effective for improving model performance, including MixUp and CutMix.

For the network architecture, despite numerous segmentation networks having been proposed in recent years, the classical U-Net-like architectures, such as U2Net and nnUNet, remain highly competitive in segmentation tasks. This finding is in accordance with the winning methods in other recent segmentation challenges.[35–37] In the classification task, EfficientNet has been used by many teams and exhibited remarkable performance. Additionally, ensemble methods were widely adopted by the leading teams, although with varying ensembling strategies. One method involves integrating predictions from distinct network architectures, while another incorporates predictions generated by the identical network architecture but fed with multiple transformed versions of the same inference image as input. In terms of accuracy, both ensemble strategies have the potential to achieve SOTA performance.

For the loss function, the dice coefficient is used as the evaluation metric in the segmentation task, with all three leading teams using the dice loss to supervise learning. This is consistent with the previous research, which demonstrated the effectiveness of the metric-sensitive loss functions in improving the corresponding metric scores.[38] In addition, a combination of different loss functions is effective in improving model performance, with the dominant combinations including dice loss paired with either cross-entropy loss or focal loss. In terms of the optimizer, AdamW, which performs L2 regularization for larger weights to further improve the training of the model, was the most commonly used by these teams in both segmentation and classification tasks.

For the post-processing, in disease-grading tasks, segmentation of the lesion area can help improve the performance of disease grading. For instance, in DR grading, the presence of NV corresponds to the PDR grade. However, in certain images, NV regions are often small and are difficult to detect, potentially leading the model to misclassify the image as NPDR. In such cases, if a lesion segmentation model has detected the NV region, the image can be corrected to PDR grade. The top two methods in the DR-grading task both leveraged the lesion segmentation results to correct the predicted DR grade made by the classification model, resulting in an improvement in classification performance.

Limitations of the study

In the domain of DR diagnosis, PDR stands as the most severe grade, carrying a high risk of causing severe vision impairment and even blindness. The employment of UW-OCTA imaging has demonstrated a superior PDR detection rate in comparison to clinical examination.[16] Consequently, a substantial pool of PDR images for training becomes essential for deep learning methods to extract generalized features of PDR lesions. However, within this challenge dataset, only about 11% (70 images) fall into the PDR category in the training set, hindering the ability of the methods to extract effective PDR features and achieve generalization in clinical practice. In addition, the performance of the NPA segmentation still lags behind that reported in some existing studies. This performance gap needs to be further investigated from three primary angles. First, larger datasets, as used in some studies,[39,40] may improve model performance. Second, including FAZ in NPA segmentation, as seen in these works,[39–41] is crucial. The fixed position and distinct patterns of FAZ in the image simplify its segmentation, thereby improving the overall segmentation performance. Finally, incorporating a variety of retinal layers into the network,[39–41] particularly by integrating data from multiple retinal layers such as the superficial vascular complex, intermediate capillary plexus, and deep capillary plexus, allows the network to extract richer features, further improving the accuracy of NPA segmentation.

Moreover, our dataset does not contain meta-information about the images, such as age, gender, height, and medical history. This lack prevents us from providing statistics on the number of eyes and patients within the dataset. In addition, the Grand Challenge platform deployed in this challenge offers two submission options: one is algorithm submission and another is result submission. In comparison, result submission boasts the advantage of being free for organizers, consuming fewer

computational resources of the platform, and presenting a simple online evaluation process for participants. Thus, we have opted for result submission as the avenue for our challenge submission. Nonetheless, a notable drawback lies in the fact that participants could have access to the test images without annotations. This opens the door for participants to optimize network parameters using the test images, potentially introducing biases into the output of the algorithm.

Future work

In our future efforts for the DRAC challenge, we intend to expand the dataset, providing more images for model training and validation. Additionally, we expect to include meta-information that can facilitate subgroup analysis, enabling us to assess model biases across different populations, such as gender and age. Moreover, we will make the DRAC dataset accessible to the community through the DRAC website, with the hope that it will prove highly valuable for researchers addressing various topics in this field. Beyond the end of this challenge, we will also maintain an open post-challenge submission system, see in https://drac22.grand-challenge.org/post-challenge-submission, encouraging the evaluation of innovative solutions and driving progress in the domain of automated DR analysis.

Conclusion

The DRAC challenge held at MICCAI 2022 provides a benchmark DRAC dataset and evaluation framework for automatic DR analysis from UW-OCTA images, including the tasks of DR lesion segmentation, image quality assessment, and DR grading. With numerous participants from academia and industry worldwide, the challenge offers diverse solutions and comparable diagnostic results to benefit ophthalmologists engaged in DR analysis. These solutions are described in detail in the MICCAI Challenge Proceedings, and many teams, including the top three teams in each task, have open-sourced their code, which can significantly accelerate methodological developments in the research community. We thoroughly summarized and discussed the algorithms and results from participating teams in this paper. These algorithms hold the potential to be integrated into future computer-assisted automatic diagnostic systems for DR, which can help reduce the burden on healthcare workers and improve the accuracy of DR diagnosis. Nevertheless, ongoing research efforts are still needed to improve the model and realize a clinically applicable diagnostic system for DR. To date, the challenge website remains open for post-challenge submissions, with the aim of providing a sustainable benchmarking and evaluation platform for the research community.

EXPERIMENTAL PROCEDURES

Resource availability

Lead contact

Requests for further information and resources should be directed to the lead contact, Bin Sheng (shengbin@sjtu.edu.cn).

Materials availability

This study did not generate any new materials.

Data and code availability

The DRAC dataset has been deposited to the Zenodo data repository under https://doi.org/10.5281/zenodo.10280358.[42] The code for methods A1, B1, and C1 has been archived at Zenodo under https://doi.org/10.5281/zenodo.10254200.[43] The code for methods A2, B2, and C2 has been archived at Zenodo under https://doi.org/10.5281/zenodo.10212156.[44] The code for method A3 has been archived at Zenodo under https://doi.org/10.5281/zenodo.10254707.[45] The code for method B3 has been archived at Zenodo under https://doi.org/10.5281/zenodo.10210181.[46] The code for method C3 has been archived at Zenodo under https://doi.org/10.5281/zenodo.10209637.[47]

Ethics statement

The study adhered to the guidelines of the Helsinki Declaration and had the approval of the Ethics Committee of Shanghai Sixth People's Hospital(2019-KY-052(K)-(3)), Shanghai, China. All patients signed written informed consent for participation. According to the Common Rule by the Department of Health and Human Services, this informed consent includes that eight elements of information about the research study be provided to the patient or his or her legally authorized representative.[48] These elements include a statement that the study is investigational along with a description of the research and its objectives, a description of foreseeable risks, a description of foreseeable benefits to participants as well as to others, information about reasonable alternatives, a statement clarifying the implications of research participation for the subject's confidentiality, a statement about compensation if injury occurs (for investigations involving more than minimal risk), information about how the subject can obtain answers to pertinent research questions, and a statement about the voluntary nature of study participation and the subject's right to withdraw.

Evaluation metrics

There are three leaderboards in the challenge website, and each task corresponds to a leaderboard. In the task of DR lesion segmentation, DSC is used for the algorithm evaluation and ranking in segmentation task. In case of a tie, Intersection of Union (IoU) is used as an auxiliary ranking metric. The DSC and IoU are calculated as follows.

$$\mathrm{DSC} = \frac{2|G \cap P|}{|G| + |P|} \quad \text{(Equation 1)}$$

$$\mathrm{IoU} = \frac{|G \cap P|}{|G \cup P|} \quad \text{(Equation 2)}$$

where G and P are the ground-truth mask and predicted mask, respectively. The metric is calculated for each class independently, and the results are then averaged.

For the tasks of image quality assessment and DR grading, quadratic weighted kappa is used for the algorithm evaluation and ranking. In case of a tie, area under the receiver operating characteristic curve (AUC) is used as an auxiliary ranking metric. Considering that this is a multi-class task, the macro averaging[49] and one-vs.-one (OVO) strategy[50] are used to calculate the AUC value. Macro averaging calculates the AUC value for each label and finds their unweighted mean. The OVO strategy computes the average AUC of all possible pairwise combinations of classes. The quadratic weighted kappa K_w is calculated as follows.

$$K_w = 1 - \frac{\sum_{i,j} \mathrm{w}_{i,j} O_{i,j}}{\sum_{i,j} \mathrm{w}_{i,j} E_{i,j}} \quad \text{(Equation 3)}$$

where O and E are the histogram matrix and expected matrix, respectively, with the size of $\mathrm{N} \times \mathrm{N}$. The weighted matrix w is defined by $\mathrm{w}_{i,j} = \frac{(i-j)^2}{(\mathrm{N}-1)^2}$, where i and j denote the actual value and the predicted value, respectively. N is the number of classes.

SUPPLEMENTAL INFORMATION

Supplemental information can be found online at https://doi.org/10.1016/j.patter.2024.100929.

ACKNOWLEDGMENTS

We thank all the investigators and participants in this study. This study was supported by the National Key Research and Development Program of China

CellPress
OPEN ACCESS

(2022YFC2502800, 2022YFA1004804, and 2022YFC2407000), the Shanghai Municipal Key Clinical Specialty, Shanghai Research Center for Endocrine and Metabolic Diseases (2022ZZ01002) and the Chinese Academy of Engineering (2022-XY-08), National Natural Science Foundation of China (823881007, 62272298, 82270907, 82022012, and 62077037), Innovative research team of high-level local universities in Shanghai (SHSMU-ZDCX20212700), the Interdisciplinary Program of Shanghai Jiao Tong University (YG2023LC11, YG2022ZD007, and YG2022QN089), and the College-level Project Fund of Shanghai Sixth People's Hospital (ynlc201909).

AUTHOR CONTRIBUTIONS

Conceptualization, B.Q., H. Chen, X.W., Z.G., T.L., H.L., Y.C.T., T.Y.W., and B.S.; methodology, B.Q., H. Chen, X.W., Z.G., T.L., Y. Wen, H. Che, G.K., J.K., S.C., S.S., F.K., M.U., J.H., R.F., Y.L., M.E.H.D., C.Y.C., D.Y., Q.W., P.Z., X.Y., Y.C., G.S.W.T., and W.J.; formal analysis, B.Q., H. Chen, X.W., Z.G., Y.J., Y. Wu, and T.L.; writing – original draft, B.Q., H. Chen, X.W., Z.G., and T.L.; writing – review & editing, C.Y.C., D.Y., Q.W., P.Z., X.Y., Y.C., G.S.W.T., W.J., H.L., Y.C.T., T.Y.W., and B.S.

DECLARATION OF INTERESTS

The authors declare no competing interests.

Received: September 25, 2023
Revised: December 9, 2023
Accepted: January 15, 2024
Published: February 8, 2024

REFERENCES

1. Reichel, E., and Salz, D. (2015). Diabetic retinopathy screening. In Managing Diabetic Eye Disease in Clinical Practice, R.P. Singh, ed. (Springer International Publishing), pp. 25–38. https://doi.org/10.1007/978-3-319-08329-2_3.
2. (2017). IDF Diabetes Atlas. (Brussels, Belgium: International Diabetes Federation (IDF)), p. 147.
3. Wang, W., and Lo, A.C.Y. (2018). Diabetic Retinopathy: Pathophysiology and Treatments. Int. J. Mol. Sci. *19*, 1816. https://doi.org/10.3390/ijms19061816.
4. American Diabetes Association (2020). 10. Cardiovascular Disease and Risk Management: Standards of Medical Care in Diabetes—2020. Diabetes Care *43*, S111–S134. https://doi.org/10.2337/dc20-S010.
5. Guan, Z., Li, H., Liu, R., Cai, C., Liu, Y., Li, J., Wang, X., Huang, S., Wu, L., Liu, D., et al. (2023). Artificial intelligence in diabetes management: Advancements, opportunities, and challenges. Cell Rep. Med. *4*, 101213. https://doi.org/10.1016/j.xcrm.2023.101213.
6. Jones, S., and Edwards, R.T. (2010). Diabetic retinopathy screening: a systematic review of the economic evidence. Diabet. Med. *27*, 249–256. https://doi.org/10.1111/j.1464-5491.2009.02870.x.
7. Lin, S., Ramulu, P., Lamoureux, E.L., and Sabanayagam, C. (2016). Addressing risk factors, screening, and preventative treatment for diabetic retinopathy in developing countries: a review. Clin. Exp. Ophthalmol. *44*, 300–320. https://doi.org/10.1111/ceo.12745.
8. Wang, L.Z., Cheung, C.Y., Tapp, R.J., Hamzah, H., Tan, G., Ting, D., Lamoureux, E., and Wong, T.Y. (2017). Availability and variability in guidelines on diabetic retinopathy screening in Asian countries. Br. J. Ophthalmol. *101*, 1352–1360. https://doi.org/10.1136/bjophthalmol-2016-310002.
9. Ting, D.S.W., Cheung, G.C.M., and Wong, T.Y. (2016). Diabetic retinopathy: global prevalence, major risk factors, screening practices and public health challenges: a review. Clin. Exp. Ophthalmol. *44*, 260–277. https://doi.org/10.1111/ceo.12696.
10. Dai, L., Wu, L., Li, H., Cai, C., Wu, Q., Kong, H., Liu, R., Wang, X., Hou, X., Liu, Y., et al. (2021). A deep learning system for detecting diabetic retinopathy across the disease spectrum. Nat. Commun. *12*, 3242. https://doi.org/10.1038/s41467-021-23458-5.
11. Jelinek, H., and Cree, M.J. (2009). Automated Image Detection of Retinal Pathology (Crc Press).
12. Li, Z., Keel, S., Liu, C., He, Y., Meng, W., Scheetz, J., Lee, P.Y., Shaw, J., Ting, D., Wong, T.Y., et al. (2018). An Automated Grading System for Detection of Vision-Threatening Referable Diabetic Retinopathy on the Basis of Color Fundus Photographs. Diabetes Care *41*, 2509–2516. https://doi.org/10.2337/dc18-0147.
13. Gulshan, V., Peng, L., Coram, M., Stumpe, M.C., Wu, D., Narayanaswamy, A., Venugopalan, S., Widner, K., Madams, T., Cuadros, J., et al. (2016). Development and Validation of a Deep Learning Algorithm for Detection of Diabetic Retinopathy in Retinal Fundus Photographs. JAMA *316*, 2402–2410. https://doi.org/10.1001/jama.2016.17216.
14. Attaran-Rezaei, K., Saraf, S., Zhang, Q., Wang, F., and Wang, R.K. (2019). Ultra-wide field OCTA for evaluation of different stages of diabetic retinopathy. Investigative Ophthalmology and Visual Science *60*, 3017.
15. Zhang, Q., Rezaei, K.A., Saraf, S.S., Chu, Z., Wang, F., and Wang, R.K. (2018). Ultra-wide optical coherence tomography angiography in diabetic retinopathy. Quant. Imag. Med. Surg. *8*, 743–753. https://doi.org/10.21037/qims.2018.09.02.
16. Khalid, H., Schwartz, R., Nicholson, L., Huemer, J., El-Bradey, M.H., Sim, D.A., Patel, P.J., Balaskas, K., Hamilton, R.D., Keane, P.A., and Rajendram, R. (2021). Widefield optical coherence tomography angiography for early detection and objective evaluation of proliferative diabetic retinopathy. Br. J. Ophthalmol. *105*, 118–123. https://doi.org/10.1136/bjophthalmol-2019-315365.
17. Cui, Y., Zhu, Y., Wang, J.C., Lu, Y., Zeng, R., Katz, R., Vingopoulos, F., Le, R., Laíns, I., Wu, D.M., et al. (2021). Comparison of widefield swept-source optical coherence tomography angiography with ultra-widefield colour fundus photography and fluorescein angiography for detection of lesions in diabetic retinopathy. Br. J. Ophthalmol. *105*, 577–581. https://doi.org/10.1136/bjophthalmol-2020-316245.
18. Pichi, F., Smith, S.D., Abboud, E.B., Neri, P., Woodstock, E., Hay, S., Levine, E., and Baumal, C.R. (2020). Wide-field optical coherence tomography angiography for the detection of proliferative diabetic retinopathy. Graefes Arch. Clin. Exp. Ophthalmol. *258*, 1901–1909. https://doi.org/10.1007/s00417-020-04773-x.
19. Kim, K., In You, J., Park, J.R., Kim, E.S., Oh, W.-Y., and Yu, S.-Y. (2021). Quantification of retinal microvascular parameters by severity of diabetic retinopathy using wide-field swept-source optical coherence tomography angiography. Graefes Arch. Clin. Exp. Ophthalmol. *259*, 2103–2111. https://doi.org/10.1007/s00417-021-05099-y.
20. Zhu, Y., Cui, Y., Wang, J.C., Lu, Y., Zeng, R., Katz, R., Wu, D.M., Eliott, D., Vavvas, D.G., Husain, D., et al. (2020). Different Scan Protocols Affect the Detection Rates of Diabetic Retinopathy Lesions by Wide-Field Swept-Source Optical Coherence Tomography Angiography. Am. J. Ophthalmol. *215*, 72–80. https://doi.org/10.1016/j.ajo.2020.03.004.
21. Niemeijer, M., van Ginneken, B., Cree, M.J., Mizutani, A., Quellec, G., Sanchez, C.I., Zhang, B., Hornero, R., Lamard, M., Muramatsu, C., et al. (2010). Retinopathy Online Challenge: Automatic Detection of Microaneurysms in Digital Color Fundus Photographs. IEEE Trans. Med. Imag. *29*, 185–195. https://doi.org/10.1109/TMI.2009.2033909.
22. Porwal, P., Pachade, S., Kokare, M., Deshmukh, G., Son, J., Bae, W., Liu, L., Wang, J., Liu, X., Gao, L., et al. (2020). IDRiD: Diabetic Retinopathy – Segmentation and Grading Challenge. Med. Image Anal. *59*, 101561. https://doi.org/10.1016/j.media.2019.101561.
23. Liu, R., Wang, X., Wu, Q., Dai, L., Fang, X., Yan, T., Son, J., Tang, S., Li, J., Gao, Z., et al. (2022). DeepDRiD: Diabetic Retinopathy—Grading and Image Quality Estimation Challenge. Patterns *3*, 100512. https://doi.org/10.1016/j.patter.2022.100512.
24. Sun, Z., Yang, D., Tang, Z., Ng, D.S., and Cheung, C.Y. (2021). Optical coherence tomography angiography in diabetic retinopathy: an updated review. Eye *35*, 149–161. https://doi.org/10.1038/s41433-020-01233-y.
25. Nicholson, L., Ramu, J., Chan, E.W., Bainbridge, J.W., Hykin, P.G., Talks, S.J., and Sivaprasad, S. (2019). Retinal Nonperfusion Characteristics on Ultra-Widefield Angiography in Eyes With Severe Nonproliferative

Diabetic Retinopathy and Proliferative Diabetic Retinopathy. JAMA Ophthalmol. *137*, 626–631. https://doi.org/10.1001/jamaophthalmol.2019.0440.

26. Kawai, K., Uji, A., Miyazawa, T., Yamada, T., Amano, Y., Miyagi, S., Seo, R., Miyata, M., Kadomoto, S., and Tsujikawa, A. (2021). Prevention of Image Quality Degradation in Wider Field Optical Coherence Tomography Angiography Images Via Image Averaging. Transl. Vis. Sci. Technol. *10*, 16. https://doi.org/10.1167/tvst.10.13.16.
27. Wang, Y., Shen, Y., Yuan, M., Xu, J., Yang, B., Liu, C., Cai, W., Cheng, W., and Wang, W. (2021). A Deep Learning-based Quality Assessment and Segmentation System with a Large-scale Benchmark Dataset for Optical Coherence Tomographic Angiography Image. Preprint at arXiv. https://doi.org/10.48550/arXiv.2107.10476.
28. World Health Organization (2006). Prevention of Blindness from Diabetes Mellitus: Report of a WHO Consultation in Geneva, Switzerland (World Health Organization), pp. 9–11.
29. Maier-Hein, L., Reinke, A., Kozubek, M., Martel, A.L., Arbel, T., Eisenmann, M., Hanbury, A., Jannin, P., Müller, H., Onogur, S., et al. (2020). BIAS: Transparent reporting of biomedical image analysis challenges. Med. Image Anal. *66*, 101796. https://doi.org/10.1016/j.media.2020.101796.
30. Sheng, B., and Aubreville, M. (2023). Mitosis Domain Generalization and Diabetic Retinopathy Analysis: MICCAI Challenges MIDOG 2022 and DRAC 2022, Held in Conjunction with MICCAI 2022, Singapore, September 18–22, 2022, Proceedings (Springer Nature Switzerland). https://doi.org/10.1007/978-3-031-33658-4.
31. Wiesenfarth, M., Reinke, A., Landman, B.A., Eisenmann, M., Saiz, L.A., Cardoso, M.J., Maier-Hein, L., and Kopp-Schneider, A. (2021). Methods and open-source toolkit for analyzing and visualizing challenge results. Sci. Rep. *11*, 2369. https://doi.org/10.1038/s41598-021-82017-6.
32. Ganaie, M.A., Hu, M., Malik, A.K., Tanveer, M., and Suganthan, P.N. (2022). Ensemble deep learning: A review. Eng. Appl. Artif. Intell. *115*, 105151. https://doi.org/10.1016/j.engappai.2022.105151.
33. Xie, F., Fan, H., Li, Y., Jiang, Z., Meng, R., and Bovik, A. (2017). Melanoma Classification on Dermoscopy Images Using a Neural Network Ensemble Model. IEEE Trans. Med. Imag. *36*, 849–858. https://doi.org/10.1109/TMI.2016.2633551.
34. Khened, M., Kollerathu, V.A., and Krishnamurthi, G. (2019). Fully convolutional multi-scale residual DenseNets for cardiac segmentation and automated cardiac diagnosis using ensemble of classifiers. Med. Image Anal. *51*, 21–45. https://doi.org/10.1016/j.media.2018.10.004.
35. Ma, J., Zhang, Y., Gu, S., An, X., Wang, Z., Ge, C., Wang, C., Zhang, F., Wang, Y., Xu, Y., et al. (2022). Fast and Low-GPU-memory abdomen CT organ segmentation: The FLARE challenge. Med. Image Anal. *82*, 102616. https://doi.org/10.1016/j.media.2022.102616.
36. Heller, N., Isensee, F., Maier-Hein, K.H., Hou, X., Xie, C., Li, F., Nan, Y., Mu, G., Lin, Z., Han, M., et al. (2021). The state of the art in kidney and kidney tumor segmentation in contrast-enhanced CT imaging: Results of the KiTS19 challenge. Med. Image Anal. *67*, 101821. https://doi.org/10.1016/j.media.2020.101821.
37. Oreiller, V., Andrearczyk, V., Jreige, M., Boughdad, S., Elhalawani, H., Castelli, J., Vallières, M., Zhu, S., Xie, J., Peng, Y., et al. (2022). Head and neck tumor segmentation in PET/CT: The HECKTOR challenge. Med. Image Anal. *77*, 102336. https://doi.org/10.1016/j.media.2021.102336.
38. Bertels, J., Eelbode, T., Berman, M., Vandermeulen, D., Maes, F., Bisschops, R., and Blaschko, M.B. (2019). Optimizing the Dice Score and Jaccard Index for Medical Image Segmentation: Theory and Practice. In Medical Image Computing and Computer Assisted Intervention – MICCAI 2019 Lecture Notes in Computer Science, D. Shen, T. Liu, T.M. Peters, L.H. Staib, C. Essert, S. Zhou, P.-T. Yap, and A. Khan, eds. (Springer International Publishing), pp. 92–100. https://doi.org/10.1007/978-3-030-32245-8_11.
39. Guo, Y., Hormel, T.T., Gao, L., You, Q., Wang, B., Flaxel, C.J., Bailey, S.T., Choi, D., Huang, D., Hwang, T.S., and Jia, Y. (2021). Quantification of Nonperfusion Area in Montaged Widefield OCT Angiography Using Deep Learning in Diabetic Retinopathy. Ophthalmol. Sci. *1*, 100027. https://doi.org/10.1016/j.xops.2021.100027.
40. Wang, J., Hormel, T.T., You, Q., Guo, Y., Wang, X., Chen, L., Hwang, T.S., and Jia, Y. (2020). Robust non-perfusion area detection in three retinal plexuses using convolutional neural network in OCT angiography. Biomed. Opt Express *11*, 330–345. https://doi.org/10.1364/BOE.11.000330.
41. Guo, Y., Camino, A., Wang, J., Huang, D., Hwang, T.S., and Jia, Y. (2018). MEDnet, a neural network for automated detection of avascular area in OCT angiography. Biomed. Opt Express *9*, 5147–5158. https://doi.org/10.1364/BOE.9.005147.
42. DRAC Challenge (2023). Diabetic Retinopathy Analysis Challenge Dataset. Zenodo. https://doi.org/10.5281/zenodo.10280359.
43. Kwon, G., and Kim, J. (2023). anears-vuno/DRAC22_MICCAI_FAI: v1.0.0 (v1.0.0). Zenodo. https://doi.org/10.5281/zenodo.10254200.
44. Seoyoung, J., and J-hyup. (2023). KT-biohealth/DRAC22: KT-biohealth/DRAC22_final (DRAC22). Zenodo. https://doi.org/10.5281/zenodo.10212156.
45. Krause, F. (2023). flixmk/DRAC22-JKU: DRAC22-JKU (v0.1.0). Zenodo. https://doi.org/10.5281/zenodo.10254707.
46. Hou, J., and Xiao. (2023). FDU-VTS/DRAC: Code Release 1.0.0 (v1.0.0). Zenodo. https://doi.org/10.5281/zenodo.10210181.
47. LI, Y., and EL HABIB DAHO, M. (2023). Mostafa-EHD/Diabetic_Retinopathy_OCTA: important release (v1.0). Zenodo. https://doi.org/10.5281/zenodo.10209637.
48. del Carmen, M.G., and Joffe, S. (2005). Informed Consent for Medical Treatment and Research: A Review. Oncol. *10*, 636–641. https://doi.org/10.1634/theoncologist.10-8-636.
49. Pedregosa, F., Varoquaux, G., Gramfort, A., Michel, V., Thirion, B., Grisel, O., Blondel, M., Prettenhofer, P., Weiss, R., Dubourg, V., et al. Scikit-learn: Machine Learning in Python. MACHINE LEARNING IN PYTHON.
50. Hand, D.J., and Till, R.J. (2001). A Simple Generalisation of the Area Under the ROC Curve for Multiple Class Classification Problems. Mach. Learn. *45*, 171–186. https://doi.org/10.1023/A:1010920819831.

Patterns, Volume *5*

Supplemental information

DRAC 2022: A public benchmark for diabetic retinopathy analysis on ultra-wide optical coherence tomography angiography images

Bo Qian, Hao Chen, Xiangning Wang, Zhouyu Guan, Tingyao Li, Yixiao Jin, Yilan Wu, Yang Wen, Haoxuan Che, Gitaek Kwon, Jaeyoung Kim, Sungjin Choi, Seoyoung Shin, Felix Krause, Markus Unterdechler, Junlin Hou, Rui Feng, Yihao Li, Mostafa El Habib Daho, Dawei Yang, Qiang Wu, Ping Zhang, Xiaokang Yang, Yiyu Cai, Gavin Siew Wei Tan, Carol Y. Cheung, Weiping Jia, Huating Li, Yih Chung Tham, Tien Yin Wong, and Bin Sheng

Supplemental information

DRAC 2022: A Public Benchmark for Diabetic Retinopathy Analysis on Ultra-Wide Optical Coherence Tomography Angiography Images

Bo Qian, Hao Chen, Xiangning Wang, Zhouyu Guan, Tingyao Li, Yixiao Jin, Yilan Wu, Yang Wen, Haoxuan Che, Gitaek Kwon, Jaeyoung Kim, Sungjin Choi, Seoyoung Shin, Felix Krause, Markus Unterdechler, Junlin Hou, Rui Feng, Yihao Li, Mostafa El Habib Daho, Dawei Yang, Qiang Wu, Ping Zhang, Xiaokang Yang, Yiyu Cai, Gavin Siew Wei Tan, Carol Y. Cheung, Weiping Jia, Huating Li, Yih Chung Tham, Tien Yin Wong, Bin Sheng

Supplemental Note S1: Summary of top three solutions

Task 1: segmentation of DR lesions

A1 (Kwon et al.) A1[1] proposed a method for DR lesion segmentation based on the U2-Net network[2]. They developed two independent U2-Net models for different lesions. One model focused on imbalanced data settings for small lesions (IRMA and NV), while the other emphasized hard example mining for NP segmentation. To prepare the input image, they normalized the pixel values to the range [0,1] and used the original resolution of 1024 $\times$ 1024 pixels as the network input. For NP segmentation, they employed a combination of weighted dice loss and auxiliary focal loss[3] as the training loss function, which effectively penalized false positive pixels and hard-to-distinguish pixels. On the other hand, for small-lesion segmentation, they used weighted dice loss and auxiliary binary cross-entropy loss during training, with a milder penalty on false positive pixels compared to the focal loss. During the test phase, they used Test-Time Augmentation (TTA) with rotation transformations for both IRMA and NV. In the case of NPA, they averaged the predictions of five independent models, each trained with a different random number seed. They also applied TTA with rotation transformations to each of these models. Post-processing techniques were used to obtain the final lesion predictions. For NP, they applied a dilation operation with a kernel size of 5 to the predicted NPA masks. For IRMA and NV masks, if a pixel was predicted to belong to both IRMA and NV, they retained the class with higher confidence. Code is available at https://github.com/vuno/DRAC22_MICCAI_FAI and has been archived at Zenodo under https://doi.org/10.5281/zenodo.10254200.[4]

A2 (Choi et al.) A2[5] introduced an ensemble model by combining ConvNext[6], SegFormer[7], and Swin Transformer[8] to develop their segmentation model. For ConvNext, they employed a combination of dice loss and cross-entropy loss during training, while for SegFormer and Swin Transformer, the combination of dice loss and focal loss was used. To improve segmentation performance, colorization and contrast limited adaptive histogram equalization (CLAHE)[9] techniques were applied. The original image size of 1024 $\times$ 1024 was retained to prevent feature loss during training. The optimization process involved using AdamW[10] as the optimizer and polyLR for learning rate adjustment strategy. Additionally, the best-performing segmentation network was utilized to generate pseudo-labels for the images in Task 3 of DR grading. These images, along with their pseudo-labels, were used as input again for the network training. Finally, the segmentation results for IRMA and NV were obtained by applying an ensemble of the three networks using the morphological logical OR operation, while the segmentation results of NPA were obtained using the logical AND operation. Code is available at https://github.com/KT-biohealth/DRAC22 and has been archived at Zenodo under https://doi.org/10.5281/zenodo.10212156.[11]

A3 (Krause et al.) A3[12] employed nnU-Net framework[13] as their segmentation model, training individual models for each class, which allowed to fine-tune and experiment specifically for each class. Throughout the training process, they utilized a combination of dice loss and cross entropy loss, using the SGD optimizer with a momentum of 0.99 and a polynomial learning rate scheduler. To ensure generalization, they applied more than 10 data augmentation techniques for the IRMA and NV classes. However, for NPA, they discovered that fewer augmentation techniques, including flip, scale, and rotation, yielded superior results. The network was trained for 200 epochs, and a 5-fold cross-validation approach was adopted. Subsequently, only the best two or three folds were ensembled to avoid negatively impacting the overall Dice score. For IRMA, they achieved better predictions by ensembling the results using the union of all predictions. This approach proved advantageous in mitigating false negative pixels associated with IRMA. As for NPA and the NV classes, a majority vote ensembling strategy was employed to generate the final predictions. Code is available at https://github.com/flixmk/DRAC22-JKU and has been archived at Zenodo under https://doi.org/10.5281/zenodo.10254707.[14]

Task 2: image quality assessment

B1 (Kwon et al.) B1[1] employed EfficientNet-b2[15] as the network architecture, initializing it with pre-trained weights from ImageNet. To address class correlation, they treated the classification task as a regression problem in which the regression labels are 0, 1 and 2 for the three classes, respectively. Further, they used the class-specific operating thresholds to produce final predictions, as shown in Equation 4, where $\hat{y}$ represents the prediction given the input x, and f_{reg} represents the regression model. To improve generalization, they used various augmentation techniques, including flip, random gamma, and sharpen. During network training, they utilized the Smooth L1 loss function and the AdamW optimizer with a fixed learning rate of 0.0002, opting not to employ a scheduler. The model underwent 150 epochs of training with a batch size of 8. During the testing phase, a five-fold cross-validation approach was used, and ensemble methods were utilized by averaging the predictions generated by the models within each fold. Additionally, they applied TTA with flip operators for each model to further improve classification performance. Code is available at https://github.com/vuno/DRAC22_MICCAI_FAI and has been archived at Zenodo under https://doi.org/10.5281/zenodo.10254200.[4]

$$\hat{y} = \begin{cases} 0, & \text{if } f_{\text{reg}}(x) < 0.54 \\ 1, & \text{if } 0.54 \leq f_{\text{reg}}(x) < 1.5 \\ 2, & \text{otherwise} \end{cases} \tag{1}$$

B2 (Choi et al.) B2[5] employed BEIT[16] and NFNet[17] to develop the classification model. They preprocessed the input images by dividing each pixel value by 255, and used both vertical and horizontal flipping as data augmentation. They utilized the cross-entropy loss function, AdamW optimizer and the learning rate scheduler of StepLR[16] during the training process. The final result was achieved by an ensemble of BEIT and NFNet models using the following equation:

$$P = 0.55 \times S(f_{BEIT}) + 0.45 \times S(f_{NFNet}) \tag{2}$$

where P is the probabilities of the predicted classes, S represents the softmax function, and f_{BEIT} and f_{NFNet} denote the outputs from the BEIT and NFNet networks, respectively. Code is available at https://github.com/KT-biohealth/DRAC22 and has been archived at Zenodo under https://doi.org/10.5281/zenodo.10212156.[11]

B3 (Hou et al.) B3[18] designed an ensemble comprising three models: Inception-V3[19], SE-ResNeXt[20], and Vision Transformer (ViT)[21]. To address the challenge of overfitting due to limited samples, the models were pre-trained on the OCTA-25K-IQA-SEG dataset[22]. Then fine-tuning was carried out on the DRAC dataset, incorporating effective data augmentation techniques like MixUp[23] and CutMix[24], etc. All images were resized to specific dimensions: 224 × 224 for SE-ResNeXt, 384 × 384 for ViT, and 512 × 512 for Inception-V3. Additionally, they normalized all images by setting the mean to 0.5 and standard deviation to 0.5. For training the networks, they employed the SGD algorithm with an initial learning rate of 0.001 and the cosine annealing learning rate schedule. Each network was trained for 100 epochs. For the ensemble, the final prediction for each OCTA image was generated by averaging the outputs of the individual models. Code is available at https://github.com/FDU-VTS/DRAC and has been archived at Zenodo under https://doi.org/10.5281/zenodo.10210181.[25]

Task 3: DR grading

C1 (Kwon et al.) C1[1] utilized EfficientNet-b2[15] as their network architecture, initializing it with pre-trained weights from ImageNet. They approached the classification task as a regression problem, where the predicted class was obtained by rounding the predicted value to the nearest integer. During network training, they applied the Smooth L1 loss function and the AdamW optimizer with a fixed learning rate of 0.0002. The model was trained for 150 epochs with a batch size of 8. During the testing phase, a five-fold cross-validation approach was used, and ensemble methods were utilized by averaging the predictions generated by the models within each fold. Additionally, they applied TTA with flip operators for each model to further improve classification performance. In terms of post-processing, they found that the DR grading model tended to ignore the NV lesion, which is a clear indicator of PDR. As a result, the model sometimes misclassified PDR as NPDR. Therefore they corrected the misclassified NPDR predictions with PDR if the NV lesion was present in the segmentation model's prediction mask. Conversely, if the segmentation model did not predict a lesion, they corrected the DR grading model predictions to normal. This post-processing pipeline demonstrated the effectiveness of improving the classification performance of their deep learning system. Code is available at https://github.com/vuno/DRAC22_MICCAI_FAI and has been archived at Zenodo under https://doi.org/10.5281/zenodo.10254200.[4]

C2 (Choi et al.) C2[5] utilized the BEIT network to develop their classification model. Various image processing techniques such as image coloring and resampling were applied to the dataset prior to training. The resampling involved randomly processing images of the minority class, including flipping, rotating, zooming, sharpening, and then repeatedly copying them until this class had the same number of images as the majority class. During training, the image was normalized by dividing each pixel value by 255, and the data was augmented by the flip operation. Also, the cross-entropy loss function, the AdamW optimizer and the learning rate scheduler of StepLR[16] were used. For the post-processing, they designed a combination of classification and segmentation results to improve the classification performance of DR grading. In this approach, each image underwent segmentation for three lesions: IRMA, NPA, and NV, using the segmentation model. The probabilities for each class were then obtained through the DR grading classification model. A specific rule was used to refine the classification results based on the segmentation results. For example, if the NV area from the segmentation model was large and the probability of PDR from the classification model was high, the final classification result would be set as PDR. Additionally, if the initial classification result was normal but the area of IRMAs was large, the final result would be changed to NPDR. If no lesion masks were present in the segmentation result, the final classification result would be set as normal. Code is available at https://github.com/KT-biohealth/DRAC22 and has been archived at Zenodo under https://doi.org/10.5281/zenodo.10212156.[11]

C3 (Li et al.) C3[26] carried out a comprehensive evaluation of the classification performance for DR grading, using19 different backbones with the ImageNet pre-trained weights. During the training process, the original image size of 1024 ×

1024 was used as input to the network, and the cross entropy was chosen as the loss function. Various data augmentation techniques, including random crop, random flip, and random rotation, were applied, along with the Adam optimizer with an initial learning rate of 0.0001 and a weight decay of 0.0001. The ExponentialLR learning strategy schedule was used with a gamma value of 0.99. Training was performed for 1000 epochs with a batch size of 4. Five-fold cross-validation was performed on each of the 19 backbones to ensure robust evaluation. Based on kappa values from the test set, the two most best-performing folds from the DenseNet121[27] and Efficientnet-b3[15] backbones were selected and fine-tuned using the entire training set. Finally, the model ensemble for DenseNet121 and Efficientnet-b3 was implemented and achieved the best classification results. Code is available at https://github.com/Mostafa-EHD/Diabetic_Retinopathy_OCTA and has been archived at Zenodo under https://doi.org/10.5281/zenodo.10209637.[28]

Supplemental Note S2: Results of the participating teams

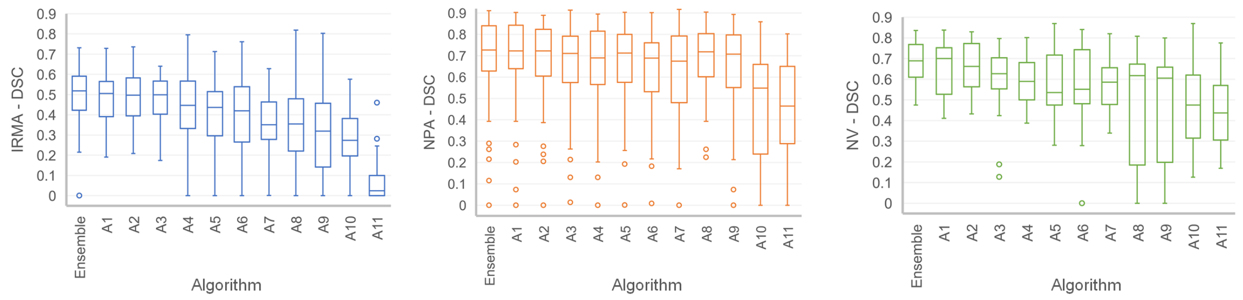

Figure S1. Box plots illustrating the DSC performance of the teams across the three lesions in DR lesion segmentation task. Ensemble represents the ensemble results of the top three algorithms.

Table S1. Quantitative results of top three teams in DR lesion segmentation task. Ensemble represents the ensemble results of the top three algorithms with a majority voting strategy.

Algorithm	IRMA				NPA				NV			
	DSC	SEN	PRE	SPE	DSC	SEN	PRE	SPE	DSC	SEN	PRE	SPE
A1	47.04	53.01	47.54	99.36	69.26	73.57	71.00	95.75	65.71	73.35	62.51	98.93
A2	48.32	57.56	47.07	99.19	67.36	74.72	69.20	94.47	65.70	77.07	60.33	98.68
A3	46.72	57.37	43.21	99.40	66.80	75.24	67.14	94.82	59.17	64.27	60.80	99.30
Ensemble	49.95	57.00	49.74	99.40	68.78	74.94	70.25	95.09	67.55	73.13	65.53	99.20

Table S2. Quantitative results of the top three algorithms in image quality assessment task. Ensemble represents the ensemble results of the top three algorithms with a majority voting strategy.

Algorithm	class	Sensitivity	Specificity	F1 score
	Poor	0.6842	0.9875	0.7536
B1	Good	0.6349	0.9627	0.6838
	Excellent	0.9763	0.7624	0.9536
	Poor	0.5789	0.9925	0.6984
B2	Good	0.7778	0.8853	0.6323
	Excellent	0.9169	0.8812	0.9392
	Poor	0.7368	0.9700	0.7179
B3	Good	0.4127	0.9733	0.5253
	Excellent	0.9822	0.6931	0.9471
	Poor	0.6842	0.9875	0.7536
Ensemble	Good	0.6349	0.9627	0.6838
	Excellent	0.9763	0.7624	0.9536

Table S3. Quantitative results of top three teams in DR grading task. Ensemble represents the ensemble results of the top three algorithms with a majority voting strategy.

Algorithm	class	Sensitivity	Specificity	F1 score
	non-DR	0.9631	0.9763	0.9721
C1	NPDR	0.8168	0.9373	0.8425
	PDR	0.7895	0.9425	0.6818
	non-DR	0.9724	0.9527	0.9679
C2	NPDR	0.7557	0.9608	0.8250
	PDR	0.8947	0.9310	0.7083
	non-DR	0.9770	0.9349	0.9636
C3	NPDR	0.8473	0.9176	0.8441
	PDR	0.5789	0.9741	0.6377
	non-DR	0.9724	0.9645	0.9724
Ensemble	NPDR	0.8473	0.9451	0.8672
	PDR	0.7895	0.9598	0.7317

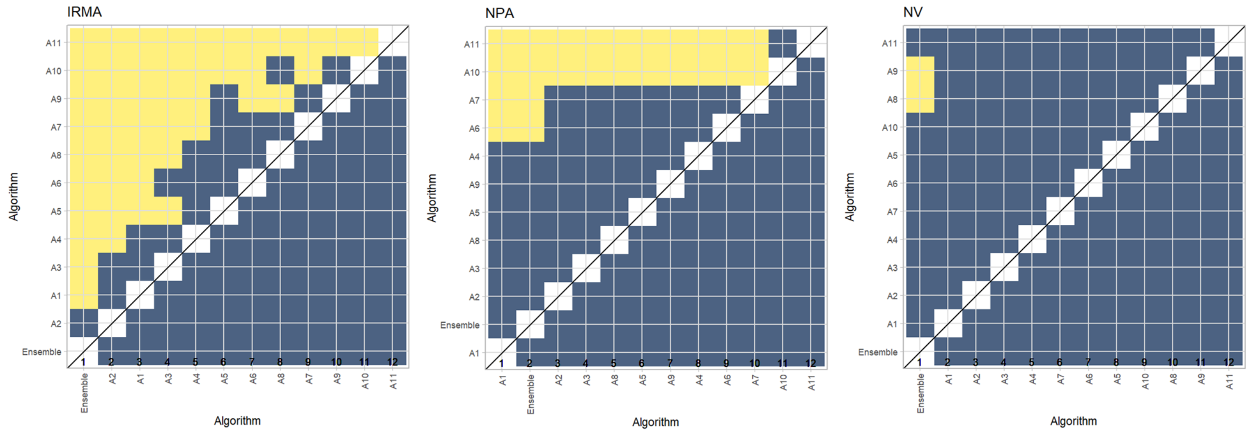

Figure S2. Significance maps depict incidence matrices of pairwise significant test results in DR lesion segmentation task for the one-sided Wilcoxon signed rank test at a 5% significance level with adjustment for multiple testing according to Holm. Yellow shading indicates that the mean DSC of the algorithm on the x-axis is significantly superior to those from the algorithm on the y-axis, and blue color indicates no significant difference.

Table S4. Summary of data augmentation techniques of top three algorithms in each task of the challenge. F, flipping; R, rotation; C, cropping; S, scaling; B, brightness; C, contrast; G, gamma; SP, sharpen; BL, blur; GD, grid distortion; CD, coarse dropout; CO, cut out; GN, gaussian noise; A, affine; MU, MixUp; CM, CutMix; TTA, test-time augmentation.

Algorithm		Data augmentation										
		F	R	C	S	B	CT	G	SP	BL	TTA	Others
	A1	✓	✓		✓	✓	✓	✓	✓	✓	✓	A/GD/CD
Task 1	A2	✓	✓									
	A3	✓	✓		✓	✓	✓	✓	✓	✓		CO/GN
	B1	✓	✓		✓	✓	✓	✓	✓	✓	✓	
Task 2	B2	✓										
	B3	✓		✓	✓	✓	✓					MU/CM
	C1	✓	✓		✓	✓	✓	✓	✓	✓	✓	CD
Task 3	C2	✓										
	C3	✓	✓	✓								

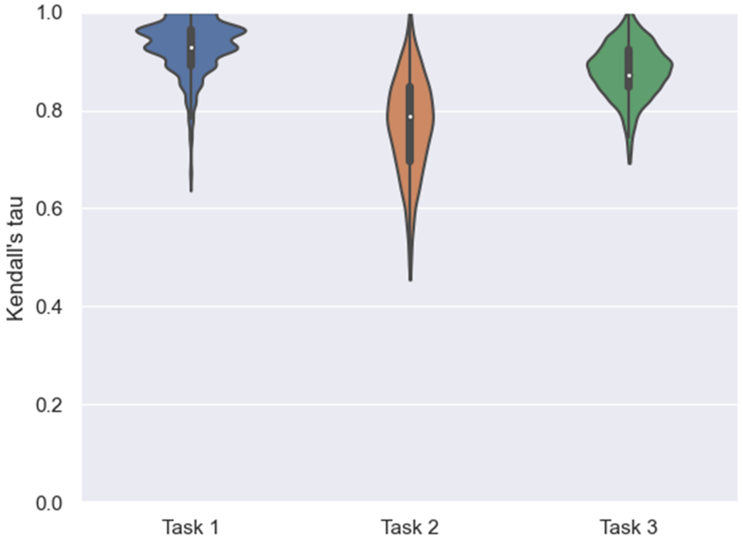

Figure S3. Violin plots of Kendall's τ for visualizing ranking stability based on bootstrapping. 1000 bootstrap samples are used for each task. The ranking list based on the full test data is compared pairwise with the ranking lists based on the individual bootstrap samples. Kendall's τ is computed for each pair of rankings, and a violin plot that simultaneously depicts a boxplot and a density plot is generated from the results.

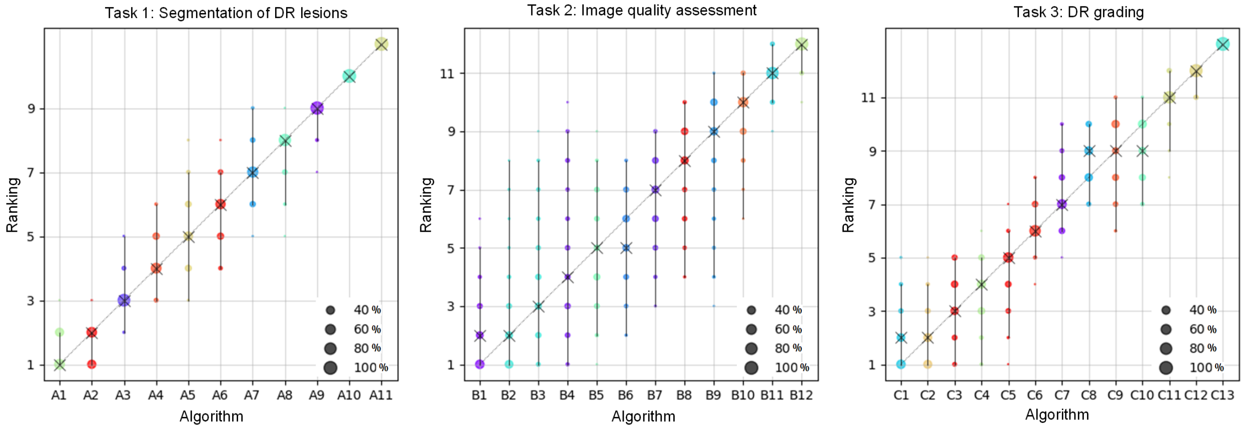

Figure S4. Blob plots for visualizing ranking stability based on bootstrap sampling. 1000 bootstrap samples are used for each task. The size of each circle is proportional to the relative frequency an algorithm obtained the corresponding rank across 1000 bootstrap samples. The median rank for each algorithm is indicated by a black cross. 95% bootstrap intervals across bootstrap samples are indicated by black lines.

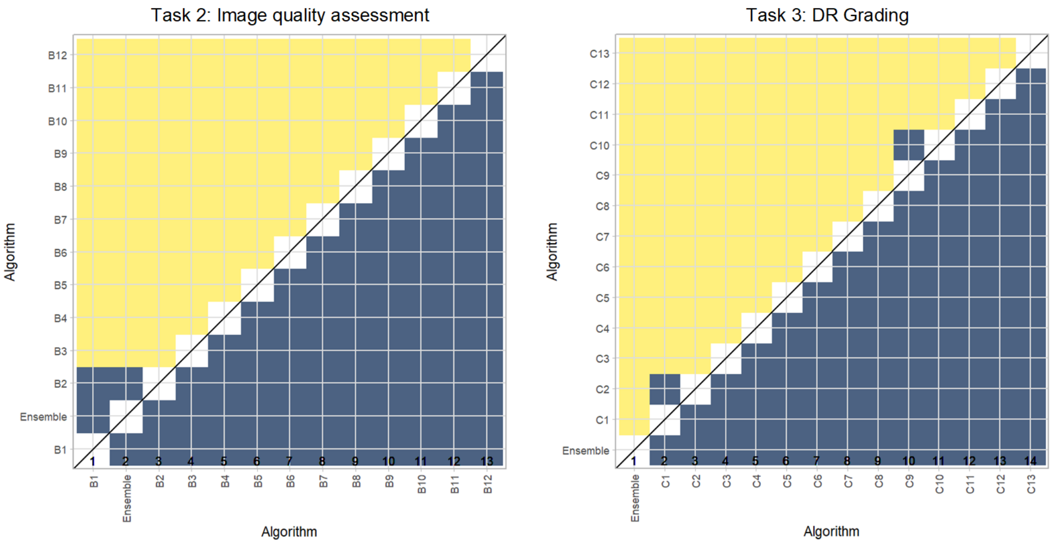

Figure S5. Significance maps depict incidence matrices of pairwise significant test results in the two classification tasks for the one-sided Wilcoxon signed rank test at a 5% significance level with adjustment for multiple testing according to Holm. Yellow shading indicates that the quadratic weighted kappa from the algorithm on the x-axis is significantly superior to those from the algorithm on the y-axis, and blue color indicates no significant difference.

References

1. Kwon, G., Kim, E., Kim, S., Bak, S., Kim, M., and Kim, J. (2023). Bag of Tricks for Developing Diabetic Retinopathy Analysis Framework to Overcome Data Scarcity. In Mitosis Domain Generalization and Diabetic Retinopathy Analysis Lecture Notes in Computer Science., B. Sheng and M. Aubreville, eds. (Springer Nature Switzerland), pp. 59–73. https://doi.org/10.1007/978-3-031-33658-4_7.
2. Qin, X., Zhang, Z., Huang, C., Dehghan, M., Zaiane, O.R., and Jagersand, M. (2020). U2-Net: Going deeper with nested U-structure for salient object detection. Pattern Recognition 106, 107404. https://doi.org/10.1016/j.patcog.2020.107404.
3. Lin, T.-Y., Goyal, P., Girshick, R., He, K., and Dollar, P. (2017). Focal Loss for Dense Object Detection. In Proceedings of the IEEE international conference on computer vision, pp. 2980-2988.
4. Kwon, G., and Kim, J. (2023). anears-vuno/DRAC22_MICCAI_FAI: v1.0.0 (v1.0.0). Zenodo. https://doi.org/10.5281/zenodo.10254200.
5. Choi, S., Jeoun, B., Anh, J., Jeong, J., Choi, Y., Kwon, D., Kim, U., and Shin, S. (2023). A Vision Transformer Based Deep Learning Architecture for Automatic Diagnosis of Diabetic Retinopathy in Optical Coherence Tomography Angiography. In Mitosis Domain Generalization and Diabetic Retinopathy Analysis Lecture Notes in Computer Science., B. Sheng and M. Aubreville, eds. (Springer Nature Switzerland), pp. 135–145. https://doi.org/10.1007/978-3-031-33658-4_13.
6. Liu, Z., Mao, H., Wu, C.-Y., Feichtenhofer, C., Darrell, T., and Xie, S. (2022). A convnet for the 2020s. In Proceedings of the IEEE/CVF conference on computer vision and pattern recognition, pp. 11976-11986.
7. Xie, E., Wang, W., Yu, Z., Anandkumar, A., Alvarez, J.M., and Luo, P. (2021). SegFormer: Simple and Efficient Design for Semantic Segmentation with Transformers. In Advances in Neural Information Processing Systems (Curran Associates, Inc.), pp. 12077–12090.
8. Liu, Z., Lin, Y., Cao, Y., Hu, H., Wei, Y., Zhang, Z., Lin, S., and Guo, B. (2021). Swin Transformer: Hierarchical Vision Transformer using Shifted Windows. In 2021 IEEE/CVF International Conference on Computer Vision (ICCV) (IEEE), pp. 9992–10002. https://doi.org/10.1109/ICCV48922.2021.00986.
9. K, Z. (1994). Contrast Limited Adaptive Histogram Equalization. Graphics Gems 0, pp. 474–485.
10. Loshchilov, I., and Hutter, F. (2019). Decoupled Weight Decay Regularization. Preprint at arXiv. https://doi.org/10.48550/arXiv.1711.05101.
11. Seoyoung, Jokulsarlon, and J-hyup. (2023). KT-biohealth/DRAC22: KT-biohealth/DRAC22_final (DRAC22). Zenodo. https://doi.org/10.5281/zenodo.10212156.
12. Krause, F., Heindl, D., Jebril, H., Karner, M., and Unterdechler, M. (2023). nnU-Net Pre- and Postprocessing Strategies for UW-OCTA Segmentation Tasks in Diabetic Retinopathy Analysis. In Mitosis Domain Generalization and Diabetic Retinopathy Analysis Lecture Notes in Computer Science., B. Sheng and M. Aubreville, eds. (Springer Nature Switzerland), pp. 5–15. https://doi.org/10.1007/978-3-031-33658-4_1.
13. Isensee, F., Jaeger, P.F., Kohl, S.A.A., Petersen, J., and Maier-Hein, K.H. (2021). nnU-Net: a self-configuring method for deep learning-based biomedical image segmentation. Nat Methods 18, 203–211. https://doi.org/10.1038/s41592-020-01008-z.
14. Krause, F. (2023). flixmk/DRAC22-JKU: DRAC22-JKU (v0.1.0). Zenodo. https://doi.org/10.5281/zenodo.10254707.
15. Tan, M., and Le, Q. (2019). EfficientNet: Rethinking Model Scaling for Convolutional Neural Networks. In Proceedings of the 36th International Conference on Machine Learning (PMLR), pp. 6105–6114.
16. Bao, H., Dong, L., Piao, S., and Wei, F. (2022). BEiT: BERT Pre-Training of Image Transformers. Preprint at arXiv. https://doi.org/10.48550/arXiv.2106.08254.
17. Brock, A., De, S., Smith, S.L., and Simonyan, K. (2021). High-Performance Large-Scale Image Recognition Without Normalization. In Proceedings of the 38th International Conference on Machine Learning (PMLR), pp. 1059–1071.
18. Hou, J., Xiao, F., Xu, J., Zhang, Y., Zou, H., and Feng, R. (2023). Deep-OCTA: Ensemble Deep Learning Approaches for Diabetic Retinopathy Analysis on OCTA Images. In Mitosis Domain Generalization and Diabetic Retinopathy Analysis Lecture Notes in Computer Science., B. Sheng and M. Aubreville, eds. (Springer Nature Switzerland), pp. 74–87. https://doi.org/10.1007/978-3-031-33658-4_8.
19. Szegedy, C., Vanhoucke, V., Ioffe, S., Shlens, J., and Wojna, Z. (2016). Rethinking the Inception Architecture for Computer Vision. In Proceedings of the IEEE conference on computer vision and pattern recognition, pp. 2818–2826.

20. Xie, S., Girshick, R., Dollar, P., Tu, Z., and He, K. (2017). Aggregated Residual Transformations for Deep Neural Networks. In 2017 IEEE Conference on Computer Vision and Pattern Recognition (CVPR) (IEEE), pp. 5987–5995. https://doi.org/10.1109/CVPR.2017.634.

21. Dosovitskiy, A., Beyer, L., Kolesnikov, A., Weissenborn, D., Zhai, X., Unterthiner, T., Dehghani, M., Minderer, M., Heigold, G., Gelly, S., et al. (2021). An Image is Worth 16x16 Words: Transformers for Image Recognition at Scale. Preprint at arXiv. https://doi.org/10.48550/arXiv.2010.11929.

22. Wang, Y., Shen, Y., Yuan, M., Xu, J., Yang, B., Liu, C., Cai, W., Cheng, W., and Wang, W. (2021). A Deep Learning-based Quality Assessment and Segmentation System with a Large-scale Benchmark Dataset for Optical Coherence Tomographic Angiography Image. Preprint at arXiv. https://doi.org/10.48550/arXiv.2107.10476.

23. Zhang, H., Cisse, M., Dauphin, Y.N., and Lopez-Paz, D. (2018). mixup: Beyond Empirical Risk Minimization. Preprint at arXiv. https://doi.org/10.48550/arXiv.1710.09412.

24. Yun, S., Han, D., Chun, S., Oh, S.J., Yoo, Y., and Choe, J. (2019). CutMix: Regularization Strategy to Train Strong Classifiers With Localizable Features. In 2019 IEEE/CVF International Conference on Computer Vision (ICCV) (IEEE), pp. 6022–6031. https://doi.org/10.1109/ICCV.2019.00612.

25. Hou, J., and Xiao. (2023). FDU-VTS/DRAC: Code Release 1.0.0 (v1.0.0). Zenodo. https://doi.org/10.5281/zenodo.10210181.

26. Li, Y., Zeghlache, R., Brahim, I., Xu, H., Tan, Y., Conze, P.-H., Lamard, M., Quellec, G., and El Habib Daho, M. (2023). Segmentation, Classification, and Quality Assessment of UW-OCTA Images for the Diagnosis of Diabetic Retinopathy. In Mitosis Domain Generalization and Diabetic Retinopathy Analysis Lecture Notes in Computer Science., B. Sheng and M. Aubreville, eds. (Springer Nature Switzerland), pp. 146–160. https://doi.org/10.1007/978-3-031-33658-4_14.

27. Huang, G., Liu, Z., Van Der Maaten, L., and Weinberger, K.Q. (2017). Densely Connected Convolutional Networks. In 2017 IEEE Conference on Computer Vision and Pattern Recognition (CVPR) (IEEE), pp. 2261–2269. https://doi.org/10.1109/CVPR.2017.243.

28. LI, Y., and EL HABIB DAHO, M. (2023). Mostafa-EHD/Diabetic_Retinopathy_OCTA: important release (v1.0). Zenodo. https://doi.org/10.5281/zenodo.10209637.
